# Epidemiology of Extended-Spectrum Beta-Lactamase and Carbapenemase-Producing Enterobacterales in the Greater Mekong Subregion: A Systematic-Review and Meta-Analysis of Risk Factors Associated With Extended-Spectrum Beta-Lactamase and Carbapenemase Isolation

**DOI:** 10.3389/fmicb.2021.695027

**Published:** 2021-11-26

**Authors:** Shweta R. Singh, Alvin Kuo Jing Teo, Kiesha Prem, Rick Twee-Hee Ong, Elizabeth A. Ashley, H. Rogier van Doorn, Direk Limmathurotsakul, Paul Turner, Li Yang Hsu

**Affiliations:** ^1^Saw Swee Hock School of Public Health, National University of Singapore, Singapore, Singapore; ^2^Department of Infectious Disease Epidemiology, Centre for Mathematical Modelling of Infectious Diseases, London School of Hygiene and Tropical Medicine, London, United Kingdom; ^3^Lao-Oxford-Mahosot Hospital Wellcome Trust Research Unit, Microbiology Laboratory, Mahosot Hospital, Vientiane, Laos; ^4^Nuffield Department of Clinical Medicine, Centre for Tropical Medicine and Global Health, University of Oxford, Oxford, United Kingdom; ^5^Oxford University Clinical Research Unit, Hanoi, Vietnam; ^6^Mahidol-Oxford Tropical Medicine Research Unit, Faculty of Tropical Medicine, Mahidol University, Bangkok, Thailand; ^7^Cambodia Oxford Medical Research Unit, Angkor Hospital for Children, Siem Reap, Cambodia; ^8^Yong Loo Lin School of Medicine, National University of Singapore, Singapore, Singapore

**Keywords:** ESBL—extended-spectrum beta-lactamase, carbapenemase, Mekong, *Escherichia coli*, *Klebsiella pneumonia*, Enterobacterales

## Abstract

**Background:** Despite the rapid spread of extended-spectrum beta-lactamase (ESBL) producing-Enterobacterales (ESBL-E) and carbapenemase-producing Enterobacterales (CPE), little is known about the extent of their prevalence in the Greater Mekong Subregion (GMS). In this systematic review, we aimed to determine the epidemiology of ESBL-E and CPE in clinically significant Enterobacterales: *Escherichia coli* and *Klebsiella pneumoniae* from the GMS (comprising of Cambodia, Laos, Myanmar, Thailand, Vietnam and Yunnan province and Guangxi Zhuang region of China).

**Methods:** Following a list of search terms adapted to subject headings, we systematically searched databases: Medline, EMBASE, Scopus and Web of Science for articles published on and before October 20th, 2020. The search string consisted of the bacterial names, methods involved in detecting drug-resistance phenotype and genotype, GMS countries, and ESBL and carbapenemase detection as the outcomes. Meta-analyses of the association between the isolation of ESBL from human clinical and non-clinical specimens were performed using the “METAN” function in STATA 14.

**Results:** One hundred and thirty-nine studies were included from a total of 1,513 identified studies. Despite the heterogeneity in study methods, analyzing the prevalence proportions on log-linear model scale for ESBL producing-*E. coli* showed a trend that increased by 13.2% (95%CI: 6.1–20.2) in clinical blood specimens, 8.1% (95%CI: 1.7–14.4) in all clinical specimens and 17.7% (95%CI: 4.9–30.4) increase in carriage specimens. Under the log-linear model assumption, no significant trend over time was found for ESBL producing *K. pneumoniae* and ESBL-E specimens. CPE was reported in clinical studies and carriage studies past 2010, however a trend could not be determined because of the small dataset. Twelve studies were included in the meta-analysis of risk factors associated with isolation of ESBL. Recent antibiotic exposure was the most studied variable and showed a significant positive association with ESBL-E isolation (pooled OR: 2.9, 95%CI: 2.3–3.8) followed by chronic kidney disease (pooled OR: 4.7, 95%CI: 1.8–11.9), and other co-morbidities (pooled OR: 1.6, 95%CI: 1.2–2.9).

**Conclusion:** Data from GMS is heterogeneous with significant data-gaps, especially in community settings from Laos, Myanmar, Cambodia and Yunnan and Guangxi provinces of China. Collaborative work standardizing the methodology of studies will aid in better monitoring, surveillance and evaluation of interventions across the GMS.

## Introduction

**FIGURE 1:**
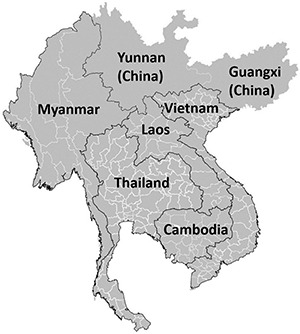
Greater Mekong Subregion (GMS) map.

Infections caused by extended-spectrum beta-lactamase (ESBL) producing Enterobacterales (ESBL-E) and carbapenemase-producing Enterobacterales (CPE) and their carriage in healthy individuals has been on the rise over the past two decades in Asia, including the Greater Mekong Sub-region (GMS) (Hsu et al., 2017; Chong et al., 2018). The GMS is a trans-national region comprising countries and territories in the Mekong river basin: Cambodia, Laos, Vietnam, Thailand, and the Yunnan province and Guangxi Zhuang autonomous region of China (Chheang, 2010; Figure 1). The GMS stretches over 2.6 million square kilometers and has a combined population of more than 340 million.^1^ The shared ecological system of GMS nations has led to overlapping health issues, especially high incidence of communicable diseases and drug resistance organisms. The GMS nations also share similar health system problems resulting from limited health investments and workforce. The GMS has undergone remarkable changes in its demographics, industrial growth and ecology (Wu et al., 2020). The region’s rapid demographic and economic expansion has put pressure on farmers facing land scarcity to intensify food production and supply by using pesticides and feed supplemented with antibiotics (Stern, 1998; Richter et al., 2015). Furthermore, much of the food in GMS comes from an integrated agriculture-aquaculture system where humans, vegetable/grain farms, livestock, and aquaculture ponds are in close proximity, which eases horizontal antibiotic-resistant gene (ARG) transfer (Zellweger et al., 2017). ARG transfer is also potentially exacerbated by lack of sanitation and adequate sewerage in low-middle income countries (LMICs) in GMS, leading to contamination of water sources and its spread (Graham et al., 2019). Furthermore, unregulated sale of antibiotics, self-medication and inappropriate prescribing of broad-spectrum antibiotics may have driven emergence of ESBL-E and CPE due to selective pressure (Om et al., 2017; Suy et al., 2019; Minh et al., 2020).

Production of ESBL and carbapenemase enzymes that hydrolyze antimicrobials is one of the most common and important mechanisms causing drug-resistance in Enterobacterales (van Hoek et al., 2011; Ruppé et al., 2015). ESBL-E can also colonize healthy individuals in the community and serve as the major reservoir for its spread (van Duin and Paterson, 2016). Surveillance of community-acquired infections has reported a widespread ESBL-mediated resistance and has shown an increasing trend for 10 years (Kanoksil et al., 2013; Fox-Lewis et al., 2018; Chang et al., 2020). Furthermore, CPE initially found in hospital settings has also been detected in carriage studies across Cambodian communities which is alarming as carbapenem is reserved as the drug of choice for severe resistant infections.

Majority studies and surveillance across provincial hospitals in individual GMS countries commonly test the phenotypic resistance (Corona and Martinez, 2013) to third-generation cephalosporins (3GC) and carbapenems (Lim et al., 2016; Fox-Lewis et al., 2018; Vu et al., 2019). Review studies conducted on Southeast Asian (SEA) articles have reported increased multidrug-resistance in Gram-negative bacteria, specifically ESBL and carbapenem resistance in Enterobacterales (Suwantarat and Carroll, 2016; Hsu et al., 2017). However, to address the increasing antimicrobial resistance (AMR) in the GMS, it is essential to attain an overview of existing evidence for directing future programs and policies, especially for monitoring and surveillance purposes. The genes encoding these enzymes are frequently found on mobile genetic elements such as plasmids and transposons that can facilitate horizontal transmission of resistance between Enterobacterales (van Hoek et al., 2011; Pierce et al., 2017). ESBLs type *bla*_CTX–M_ has been increasingly isolated after spread of the epidemic *Escherichia coli* ST131 lineage in SEA (Dunn et al., 2019). A study on SEA *Klebsiella pneumoniae* isolates also reported high rates of AMR genes highlighting the importance of genomics-based surveillance that can help standardize data for comparison between sites and identify national and regional differences (Wyres et al., 2020). Characterization of resistance mechanisms is helpful not only for clinical management, but also for tackling their spread. Furthermore, infection with ESBL-E and CPE are speculated to stem from its asymptomatic carriage in an individual’s digestive tract which has been indicated to the main reservoir from which ESBLs are derived (Tseng et al., 2018). Hence, attaining an overview of existing evidence is necessary for directing future programs and policies, especially for monitoring and surveillance. This study aimed to review ESBL-E and CPE epidemiology, their genes in both nosocomial and community settings, the diagnostic methods used in surveillance, and the risk factors associated with their isolation in GMS.

## Materials and Methods

This review study was structured following the Preferred Reporting Items for Systematic Reviews and Meta-Analysis (PRISMA) statement (Moher et al., 2011). The study was also registered with the International Prospective Register of Systematic Reviews [PROSPERO registration number: (CRD42021239409)]. Electronic databases of Medline, Embase, Scopus and Web of Science were searched using text, index, thesaurus terms for articles published on and before October 20, 2020. Relevant articles, including pre-print articles identified by snowballing, but published before March 31, 2021, were also included in the review (Vedula et al., 2011). Consistent search terms and structure were applied across these databases containing the terms specific to multidrug-resistant organisms, including ESBL-E and CPE, as presented in Table 1.

**TABLE 1:** List of search terms.

**Topic**	**Search terms**
**Context or countries in GMS**	“Greater Mekong Subregion” OR “GMS” OR “Thailand” OR “Myanmar” OR “Laos” OR “Lao PDR” OR “Cambodia” OR “Vietnam” OR “Mekong delta” OR “Yunnan Province, China” OR “Yunnan” OR “Yunnan*PRC” OR “Yunnan*China” OR “Mekong Valley” OR “Guanxi” OR “Guanxi*Zhuang”
**Bacteria**	“Enterobacteriaceae” OR “Enterobacterales” OR *“Escherichia coli”* OR “*E. coli”* OR *“Klebsiella pneumoniae*” OR *“K. pneumoniae*”
**Methods**	“Antibiotic susceptibility” OR “antibiotic sensitivity” OR “antimicrobial susceptibility” OR “antimicrobial sensitivity” OR “microbial sensitivity” OR “dis* diffusion” OR “whole genome sequencing” OR “genome sequencing” OR “bacterial genome” OR “bacterial DNA” OR “PCR” OR “Polymerase chain reaction”
**Outcome**	“multidrug resistan*” OR “multidrug-resistant organism*” OR “MDRO” OR “antimicrobial resistan*” OR “AMR” OR “antibiotic resitan*” OR “multidrug-resistant bacteria*” OR “MDRB” OR “drug resistan*” OR “Extended-spectrum beta-lactamase” OR “ESBL” OR “ESBLP-E” OR “beta-lactamase” OR “β-lactamase” OR “carbapenem-resistan*” OR “CRE” OR carbapenemase-producing” OR “CPE” OR “colistin-resistance” OR “KPC” OR “NDM” OR “MBL” OR “horizontal gene transfer” OR “lateral gene transfer” OR “molecular evolution” OR “evolution” OR “mutation”

We included studies that had collected samples from both clinical infections (clinical studies) as well as healthy individuals or hospital patients (carriage studies) and tested the samples for either ESBLs or carbapenemases in Enterobacterales, *Escherichia coli* (Ec) or *Klebsiella pneumoniae* (Kp) using either phenotypic or genotypic methods. For the prevalence of ESBLs and carbapenemases, percent of isolates detected from the given sample size were reported if the collection date and place were specified and the number of isolates was higher than 30 which is the minimum number recommended by CLSI for reporting collections of isolates (M39, 2014). Studies reporting prevalence in surveillance reports and observational studies (cross-sectional and cohort studies) were included. For cohort studies that tested the acquisition of ESBLs and carbapenemases at hospital sites, we only extracted the prevalence proportion from the specimens collected at the first time-point. Incidence rates of ESBL-E, ESBL-Ec, ESBL-Kp or CPE, CP-Ec, CP-Kp were not converted to prevalence proportion if we did not have the required information (King et al., 2014). Case-control studies were only included to extract the risk factors associated with ESBL and carbapenemase detection and not for prevalence.

Selected articles were checked for additional references missed in the initial screening of databases. English and Mandarin language studies were included in the review. Studies on only animals and environmental sites with no analysis of human samples were excluded. Two independent reviewers (SS and AT) screened the titles and abstracts, followed by full-text for the inclusion of articles in this review based on the inclusion-exclusion criteria. Interrater agreements for the screening between reviewers were high (Cohen’s kappa = 0.84). Disagreements on the inclusion of studies were discussed and resolved between reviewers.

Data from all included studies were extracted by two authors (SS and AT) in a Microsoft Excel worksheet. Study quality for observational studies reporting risk factors associated with ESBL-E or CPE isolation were assessed based on the Newcastle-Ottawa scale (Stang, 2010) independently. Data variables from individual papers included the study design, time of sample collection, country/region, participant information (clinical or carriage), sample, size, setting (community or hospital), site of sample collection (blood, urine, and all clinical specimens including stool, intra-abdominal, tracheal aspirate, wound exudate or when not specified), details of microbiological and statistical analysis among other data variables. Effect sizes (unadjusted and adjusted odds ratio) were extracted from individual studies for meta-analysis of risk factors associated with ESBL-E and CPE.

Prevalence proportions of positive ESBL and carbapenemase from the total sample size were extracted and segregated based on the participants, i.e., clinical infections or healthy individuals/hospital inpatients tested for carriage and were compiled based on the time, setting, sampling site, age of participant, place. The articles were further summarized for individual countries in the GMS. We compared the observed ESBL prevalence proportion separately for E, Ec, and Kp isolated from clinical and carriage specimens reported in the studies over time and across geographical regions. Clinical specimens were further classified as blood, urine and all clinical samples. The ESBL prevalence proportions for E, Ec, and Kp were analyzed for time trend using a log-linear model to estimate the proportion change over time (Karanika et al., 2016). For plotting on the graph, the first year of study was considered as the index year and included for studies that extended over 1 year. Similarly, for studies that extended over 2 years, the year that coincided with the mid-timepoint was designated as the index year. Studies reporting an average prevalence proportion for samples collected over the course of 5 years or more were excluded from the plot and trend test. For data sets at two different time points, prevalence proportion of latest data points were included in the plot. Besides, we pooled adjusted odds ratios and 95% confidence intervals of risk factors for each meta-analysis if data were available in two or more studies. Study heterogeneities were quantified using Chi-square statistics Q and I^2^. Due to the small number of studies analyzed and the potential biases it could have on the estimation of heterogeneities (von Hippel, 2015), we conducted all meta-analyses using both fixed and random-effects models. In the main paper, we incorporated heterogeneities among studies (*I*^2^ > 40%) into the DerSimonian-Laird random-effects model (Higgins et al., 2019). A Mantel–Haenszel fixed-effects model was presented for meta-analyses with minimal variations (*I*^2^ ≤ 40%) between studies. Meta-analyses of the association between the isolation of ESBL from clinical and non-clinical specimens and (1) previous antibiotics use, and (2) the presence of co-morbidities were performed using the “METAN” function in STATA 14 (StataCorp LP, College Station, TX, United States) (Harris et al., 2008).

## Results

**FIGURE 2:**
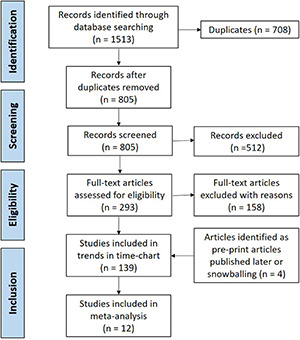
Preferred reporting items for systematic reviews and meta-analysis (PRISMA) chart displaying flow-chart of study screening and selection.

The screening flowchart, along with the number of studies, is presented according to PRISMA guidelines in Figure 2. Running the search string through databases yielded 1,513 studies, and after excluding 708 duplicate studies, 805 studies were screened for titles and abstracts. Three pre-print studies that fit the inclusion criteria but were published post the search date were included in the review (Singh et al., 2020; Aung et al., 2021; San et al., 2021). A total of 139 articles were included in the final review, with a subset of 12 studies analyzed for meta-analysis that specifically presented risk factors associated with isolation of ESBL-E and ESBL-Ec from either clinical or non-clinical (carriage) samples. Studies that reported only antibiotic susceptibility of isolates to third-generation cephalosporins or individual carbapenems (imipenem/meropenem) were excluded from this study (Vu et al., 2019).

The majority of studies in this review were conducted in Thailand (*n* = 80), followed by Vietnam (*n* = 34). Comparatively, fewer ESBL and CPE detection studies were conducted in Cambodia (*n* = 14), Myanmar (8), Laos (*n* = 3) and Yunnan and Guangxi province of China (*n* = 3). The carriage rates were reported as proportion of positive isolates of Ec, Kp or Enterobacterales (when specified as Enterobacteriaceae or Enterobacterales in the study).

**FIGURE 3:**
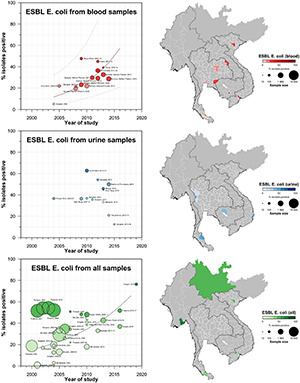
Clinical ESBL-Ec prevalence proportion in GMS. Observed ESBL prevalences, presented as proportions, of Ec isolated from clinical—blood, urine, all—specimens in the Greater Mekong Subregion over time and across geographical regions. The smallest unit of geographical regions considered, demarcated by white lines on the map, is the first-order administrative units or provinces. The size of the points corresponds to the sample size of the studies, and broken rings represent studies that do not report a sample size. Darker points and regions indicate a higher prevalence of ESBL Ec, and province with no observed data are shaded gray. If multiple studies were available for a province, the highest value is presented. For studies across multiple provinces, the ESBL Ec overall prevalences are presented for all the named provinces.

**FIGURE 4:**
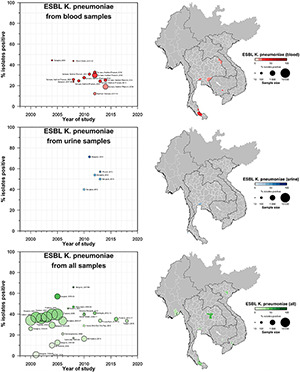
Clinical ESBL Kp prevalence proportion in GMS. Observed ESBL prevalences, presented as proportions, of Kp isolated from clinical—blood, urine, all—specimens in the Greater Mekong Subregion over time and across geographical regions. The smallest unit of geographical regions considered, demarcated by white lines on the map, is the first-order administrative units or provinces. The size of the points corresponds to the sample size of the studies, and broken rings represent studies that do not report a sample size. Darker points and regions indicate a higher prevalence of ESBL-Kp, and province with no observed data are shaded gray. If multiple studies were available for a province, the highest value is presented. For studies across multiple provinces, the ESBL Kp overall prevalences are presented for all the named provinces.

**FIGURE 5:**
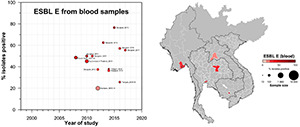
Clinical ESBL-E prevalence proportion in GMS. Observed ESBL prevalences, presented as proportions, of Enterobacterales isolated from clinical—blood specimens in the Greater Mekong Subregion over time and across geographical regions. The smallest unit of geographical regions considered, demarcated by white lines on the map, is the first-order administrative units or provinces. The size of the points corresponds to the sample size of the studies, and broken rings represent studies that do not report a sample size. Darker points and regions indicate a higher prevalence of ESBL E, and province with no observed data are shaded gray. If multiple studies were available for a province, the highest value is presented. For studies across multiple provinces, the ESBL E overall prevalences are presented for all the named provinces.

**FIGURE 6:**
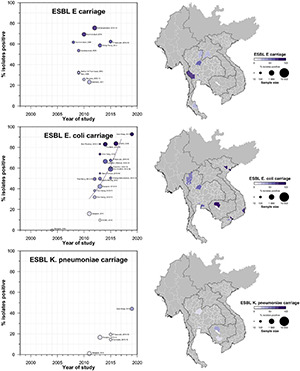
Carriage ESBL-E, Ec, and Kp prevalence proportion in GMS. Observed ESBL prevalences, presented as proportions, of E, Ec, and Kp isolated from stool or rectal swab specimens in the Greater Mekong Subregion over time and across geographical regions. The smallest unit of geographical regions considered, demarcated by white lines on the map, is the first-order administrative units or provinces. The size of the points corresponds to the sample size of the studies, and broken rings represent studies that do not report a sample size. Darker points and regions indicate a higher prevalence of ESBL Ec, and province with no observed data are shaded gray. If multiple studies were available for a province, the highest value is presented. For studies across multiple provinces, the ESBL-E, Ec, and Kp overall prevalences are presented for all the named provinces.

A total of 42 studies reported ESBL-Ec clinical isolates of which 64% (*n* = 27) were collected from a tertiary hospital setting (Supplementary Table S1). Phenotypic ESBL was detecting by double disk synergy test, MIC-E test method and VITEK-2 in isolation or combination in all studies whereas carbapenemase was detected using Modified Hodge test or mCIM method (Pierce et al., 2017; CLSI, 2020). Of the total 73 studies that reported genotype, PCR (*n* = 52) was the most common method of detection, followed by WGS (16) and combination of both (*n* = 5) (data not shown). Applying the log-linear model over the prevalence proportion observed in the past two decades, the annual prevalence of ESBL-Ec increased by 13.2% [95% CI: (6.1–20.2), *p* = 0.003] in clinical blood samples and 8.1% [95% CI:(1.7–14.4), *p* = 0.019] in all clinical samples (Figure 3), whereas no significant trend was observed for ESBL-Kp (Figure 4) and ESBL-E specimens (Figure 5). The annual ESBL-Ec prevalence in carriage samples also increased by 17.7% [95% CI: (5.0–30.4), *p* = 0.015] (Figure 6). The details of individual studies, place, specimen site, sample size and prevalence proportion of ESBL-Ec, ESBL-Kp, and ESBL-E in clinical and carriage specimens are given in Supplementary Tables S1–S3, and Supplementary Tables S4–S6, respectively.

### Thailand

Thailand contributed to the majority data from GMS, with a total of 77 studies included in this review that reported on ESBL-E or CPE or both. Surveillance of ESBL-E and CPE has been conducted in almost all individual states and regions of Thailand as presented in Figure 2.

Carriage studies conducted on healthy individuals from community settings or patients in hospital settings (but non-clinical specimens) are presented in Figure 3. Carriage rates of ESBL-Ec varied based on region and year of specimen collection. A study conducted in Kanchanaburi province (Sasaki et al., 2010; Luvsansharav et al., 2011) and populations (farmers) (Boonyasiri et al., 2014) had higher proportions of ESBL-Ec carriage (ranging from 53.6 to 75.5%) compared with other areas and populations (Supplementary Table S1), but statistical significance is uncertain owing to heterogeneity of studies. CP-Ec carriage has not been reported in any of the carriage studies conducted in Thailand.

A similar trend was observed for ESBL-Ec carriage rates in the Thailand community, with very low carriage rates observed in early 2,000 to high ESBL-Ec (>50%) in the 2010s. Only four studies reported *K. pneumoniae* carriage with a maximum prevalence rate of 19.4% reported from Phitsanulok in a 2014–2015 study (Kiddee et al., 2019). The same study reported a 3.3% carriage of CP-Kp in its sample.

Previous genotypic studies from Thailand showed the dominance of *bla*_TEM_ and *bla*_SHV,_ however, in recent studies, *bla*_CTX–M_ was found more commonly. The major ESBL genes found in ESBL-Ec isolates were *bla*_CTX–M_ (predominant subtypes –14, –15, 55) followed by *bla*_TEM–1_, *bla*_SHV–12_, *bla*_VEB–1_ and *bla*_OXA–10_. Major carbapenemase genes reported were *bla*_NDM–1_, *bla*_OXA–48_, *bla*_IMP–14_ and *bla*_KPC(–13_, _–2)_. It is interesting to note that *bla*_KPC_ genes commonly detected in Kp, was also found in Ec. The detailed list of the reported genes from each country in the GMS is presented in Supplementary Tables S7–S9.

### Vietnam

Vietnam produced the second highest number of studies following Thailand. The majority of the clinical surveillance and cross-sectional studies were conducted in the largest cities Hanoi and Ho Chi Minh city. The majority of clinical specimens comprised of blood, urine, intra-abdominal samples. The ESBL-Ec isolation proportion ranged from approximately 35% in the 2000s (Hawser et al., 2009; Huang et al., 2012) to approximately 50–60% in 2010s (Biedenbach et al., 2014; Nga et al., 2014; Chang et al., 2017; Dat et al., 2017; Hoang T. A. V et al., 2017). ESBL-Kp prevalence in clinical samples (blood, intra-abdominal and all other samples) ranged between 0% in 2004 (Jones et al., 2006) and 39.5% in 2009–2011 (Biedenbach et al., 2014). Carbapenemase prevalence in *K. pneumoniae* was reported to be 2.3% (Biedenbach et al., 2014).

Prevalence of ESBL-Ec carriage in non-clinical specimens ranged from 9.7% in healthy adults with no history of antibiotic consumption in the previous 3 months (Hoang P. H. et al., 2017) to approximately 83% in urban children from Ho Chi Minh City (Thi Quynh Nhi et al., 2018), and in farmers from Thai Binh province (Kawahara et al., 2019). The studies with the highest prevalence proportion were conducted between 2013 and 2016. A study screening patients from the urology department in a tertiary hospital in Hanoi reported that 4.85% of samples were positive for CPE (Tran et al., 2015). None of the Vietnamese studies reported ESBL-Kp carriage in healthy individuals.

Majority of the ESBL-E genes came from the *bla*_CTX–M_ family, specifically *bla*_CTX–M–15_, *bla*_CTX–M–55_, *bla*_CTX–M–27_ followed by *bla*_TEM,–1_, *bla*_SHV–12_. The most common carbapenemase genes were *bla*_NDM–1_, followed by *bla*_KPC–2_, *bla*_NDM–4_ and *bla*_OXA–48_. It is important to note that *bla*_KPC_ was detected in both Ec and Kp, similar to Thailand studies.

### Cambodia

All the clinical studies and most of the carriage studies from Cambodia were conducted in either Phnom Penh or Siem Reap. The clinical studies that reported ESBL-E evaluated samples from wound (Hout et al., 2015), urine (Ruppé et al., 2009; Moore et al., 2016), blood (Vlieghe et al., 2013), and other samples (Caron et al., 2018). The clinical studies conducted between 2010 and 2015 reported ESBL proportions that ranged from 33.7% (Caron et al., 2018) to 47.7% (Vlieghe et al., 2013). Among carriage studies, a longitudinal study reported 56.3% patients (*n* = 161/286) admitted to neonatal intensive care unit (NICU) colonized by more than 1 third-generation cephalosporin resistance (3GCR) species. Of these 3GCR isolates (*n* = 573), ESBL-Ec was detected from 96.9% isolates and ESBL-Kp was detected from 98.5% of the isolates (Turner et al., 2016). A study conducted in 2019 reported ESBL carriage rates of 92.8% in *E. coli* and 44.1% in *K. pneumoniae* isolated from healthy individuals in the community, which showed a sharp increase compared to previous studies (Singh et al., 2020). The same study also reported a CPE prevalence of 2.1% in the sample size of 290 stool samples.

Major genes identified from ESBL-Ec were *bla*_CTX–M–14_, *bla*_CTX–M–15_, and *bla*_CTX–M–55_. The two studies reporting CPE from samples collected in 2011 (Atterby et al., 2019) and 2015–2016 (Nadimpalli et al., 2019) identified *bla*_OXA–48_ and *bla*_NDM–5_, and *bla*_OXA–181_, respectively. Details of all ESBL and carbapenemase genes isolated from Cambodia is reported in Supplementary Tables S1–S3.

### Laos

A total of three studies from Laos were included. The study conducted on ESBL-Ec and ESBL-Kp prevalence was conducted in Vientiane in a tertiary hospital setting and reported a prevalence rate of 20% (Chang et al., 2020). The same study reported a fourfold increase in ESBL-Ec proportion from four (7.8%) cases in 2010 to 17 (34.7%) cases in 2014. Another study was conducted on healthy children attending a child-care facility in Vientiane and reported ESBL–E carriage of 23.2% (Stoesser et al., 2015). The ESBL genes found in *E. coli* in the study were *bla*_CTX–M–14_, *bla*_CTX–M–35_, *bla*_CTX–M–15_, *bla*_CTX–M–27_, *bla*_CTX–M–64_, *bla*_CTX–M–24_, *bla*_CTX–M–101_. In *K. pneumoniae*, the genes isolated from the study were *bla*_CTX–M–14_ and *bla*_SHV–2a_. The only study evaluating carbapenemase presence in Laotian *E. coli* isolates found *bla*_NDM_ like genes (Cusack et al., 2019).

### Myanmar

A total of six studies were included from Myanmar. All the studies were conducted in tertiary hospital settings in Yangon. The studies reported the prevalence of ESBL-Ec and ESBL-Kp to be 36.9% (Aung et al., 2018) and 33.5% (Aung et al., 2021), respectively. Carbapenemase genes were detected in the same studies, with prevalence rates of CP-Ec and CPE-Kp reported to be 8.2 and 7.3%, respectively. Furthermore, a study conducted on septicemic patients reported a CPE prevalence rate of 14% (Myat et al., 2020).

The major genes isolated from the ESBL-Ec included *bla*_CTX–M–15_, *bla*_CTX–M–14_, *bla*_CTX–M–55_, *bla*_CTX–M–27_, and major carbapenemase genes found in *E. coli* were *bla*_NDM–5_, *bla*_NDM–7_, *bla*_NDM–1_ and *bla*_OXA–181_. The only study on ESBL and CPE genes in *K. pneumoniae* reported *bla*_CTX–M–1_ and *bla*_CTX–M–9_ groups, *bla*_SHV–27_ and *bla*_NDM–5_, *bla*_NDM–7_, and *bla*_NDM–1_ (Aung et al., 2021).

### China

A total of two studies were included from the Yunnan province and Guangxi Zhuang autonomous region of China. The only prevalence study from clinical specimens that fit our inclusion criteria reported ESBL-Ec prevalence of 48.4% and was conducted in Dali Bai, Yunnan (Zhao et al., 2014). The ESBL-Ec found in these studies were *bla*_CTX–M–1_ and *bla*_CTX–M–9_ groups. *bla*_TEM–1_ and *bla*_SHV–1_ (Zhao et al., 2014; Zheng et al., 2016). The two carbapenemase genes found in the clinical samples were *bla*_NDM–1_, *bla*_IMP–4_ (Zheng et al., 2016).

## Risk Factors Associated With Extended-Spectrum Beta-Lactamase and Carbapenemase Isolation

**FIGURE 7:**
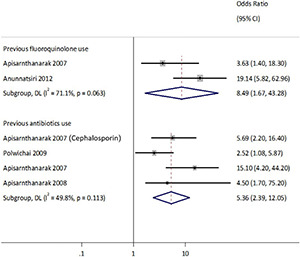
Forest plot of the risks factor associated with ESBL Enterobacterales. Carriage studies. Forest plots from the fixed-effects meta-analyses of studies showing the individual and pooled odds ratios for the association between (1) presence of comorbidities (top), (2) chronic kidney disease (mid), and (3) previous antibiotics use (bottom) and the isolation of Enterobacteriaceae (ESBL-E and CPE) from clinical and non-clinical specimens. The gray shaded box on the individual point estimates represents the relative weight of each individual study contributes to the pooled effect.

Moderate to substantial heterogeneities existed for studies that reported previous antibiotic (*Q* = 6.0, *p* = 0.11, *I*^2^ = 49.8%) and specifically fluoroquinolone use (*Q* = 3.5, *p* = 0.06, *I*^2^ = 71.1%) as presented in Figure 7. The combined analysis of eligible studies indicated that previous antibiotic use was a significant risk factor associated with the isolation of ESBL-Ec and CP-Ec [pooled odds ratio (OR): 5.4, 95% CI: 2.4–12.01], as well as other ESBL-E and CPE (pooled OR:2.9, 95%CI: 2.26–3.80) in the GMS. Presence of comorbidities (pooled OR:1.6, 95%CI: 1.2–2.2) and chronic kidney disease (pooled OR:4.7, 95%CI: 1.8–12.0) were also associated with the isolation of ESBL-E as presented in Figure 7. The forest plot based on random-effects model for ESBL-E is presented in Supplementary Figures S1, S2 and fixed-effects model for ESBL-Ec is presented in Supplementary Figure S3. A study conducted in neonates in an NGO hospital in Siem Reap reported hospital birth to be a statistically significant risk factor on multivariable analysis (aOR: 3.0, 95%CI: 1.7–5.4), while probiotic treatment appeared to be protective (Hazards ratio: 0.58, 95%CI: 0.35–0.98) (Turner et al., 2016).

Other statistically significant risk factors not included in the meta-analyses due to insufficient studies, were malignancy [adjusted OR (aOR): 3.1, 95%CI: 1.1–9.3] (Musikatavorn et al., 2011), prolonged admission to hospital (aOR: 3.1, 95%CI: 1.9–5.0) (Chaiwarith et al., 2008; Pornsinchai et al., 2015), and previous healthcare visit (aOR: 3.0, 95%CI: 1.7–5.7) (Pornsinchai et al., 2015; Turner et al., 2016). Other environmental and dietary factors that were significantly associated with ESBL-E isolation in travelers were travel history to Southeast Asia (specifically CTX-M -1 and -9 groups) (aOR: 4.3, 95%CI: 1.8–10.3) (Barreto Miranda et al., 2016), and consumption of undercooked meat (aOR: 2.1, 95%CI: 1.3–3.5) (Niumsup et al., 2018). Two studies were identified that reported the risk factors of carbapenem-resistant Enterobacterale (CRE) and CPE in hospitalized patients. A study conducted in 12 Vietnam hospitals identified healthcare-associated Infection (HAI) (aOR: 1.7, 95%CI: 1.2–2.6) and recent carbapenem use (aOR:1.8, 95%CI: 1.2–2.7) to be the risk factors associated with CRE. Another study reported independent predictors of carbapenemase in ertapenem-non-susceptible Enterobacterales isolated from intra-abdominal infections viz. positivity of the ESBL encoding allele(s) by PCR test (aOR: 11.5, 95%CI: 2.8–47.1) and sample cultured from the peritoneal space (tissue, abscess) (aOR: 3.3, 95%CI: 1.4–7.9) (Jean et al., 2018).

**FIGURE 8:**
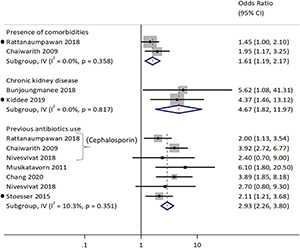
Forest plot for previous antibiotics consumption as a risk factor associated with ESBL-*E. coli.* Carriage studies. Forest plots from the random-effects meta-analyses of studies showing the individual and pooled odds ratios for the association between (1) previous fluroquinolone use (top half) and (2) previous antibiotics use (bottom half) and the isolation of *E. coli* (ESBL-Ec) from clinical and non-clinical specimens. The gray shaded box on the individual point estimates represents the relative weight of each individual study contributes to the pooled effect.

Factors that were significantly associated with ESBL-Ec isolation (Figure 8) other than the risk factors for ESBL-E included environmental risk factors of staying in a rural region (aOR: 2.04, 95%CI: 1.3–3.2) as reported in a Vietnamese study (Trung et al., 2019). A study conducted among urban Vietnamese children also reported an inverse association with *bla*_CTX–M_ gene carriage in older children (aOR: 0.97, 95%CI: 0.94–0.99) for each additional year (Thi Quynh Nhi et al., 2018).

## Discussion

This review highlighted the stark data gaps in ESBL and carbapenemase, especially from Laos, Myanmar and Cambodia in the GMS. The majority of ESBL and carbapenemase studies in these GMS countries were conducted on clinical samples obtained in tertiary hospitals from major cities (Yangon for Myanmar, Vientiane for Laos, Phnom Penh and Siem Reap for Cambodia). There was a dearth of studies from community settings in Myanmar, Yunnan and Guangxi provinces of China, highlighting the significant gap compared to Thailand and Vietnam, which had comparatively more provincial studies. The concentration of studies in hospital settings gives us minimal idea about ESBL and CPEs carriage in communities and other non-urban and rural settings. Thailand is the most researched nation and the only upper-middle-income country in GMS with a robust public health set-up and surveillance systems. The second most researched country in GMS is Vietnam. However, the heterogeneous study designs and sampling strategy makes data comparison difficult. Nevertheless, recent advances in next-generation sequencing have helped identify the common genes and sequence types from GMS, which will be beneficial in keeping track of drug-resistant mechanism in Enterobacterales (Holt et al., 2015; Matsumura et al., 2016).

Our analysis has reported an increasing trend for ESBL detection in clinical (8.1% each year) and carriage Ec isolates (17.7% each year) and that with the current high prevalence proves that ESBL is widespread and dominant in the both nosocomial and community settings. Although, we did not find any significant trend for ESBL detection in either Enterobacterales or Kp isolates, a rising trend cannot be ruled out unless we have a more consistent and homogenous data set from individual GMS nations. We also could not analyze the trend for carbapenemase given the small and uneven dataset. A meta-analysis on Carbapenem-resistant Kp had reported a high (>5%) prevalence proportion in Thailand and Vietnam and low (<1%) in Cambodia (Malchione et al., 2019). However, a study from Myanmar published in 2019 reported a high (7.3%) CPE prevalence in clinical isolates (Aung et al., 2018). Thus, accounting for missing data is essential as GMS nations except Thailand and Vietnam have fewer data points to inform prevalence proportion for the nation. Furthermore, majority of microbiology data comes from tertiary hospital settings, and it is impossible to generalize either high or low prevalence for the whole nation based on limited information.

Previous domination of ESBL type *bla*_TEM_ and *bla*_SHV_ in Thailand studies have been increasingly replaced by *bla*_CTX–M_, which has become endemic in both Ec and Kp (clinical and carriage) specimens in the entire GMS. We found *bla*_CTX–M–15_ and *bla*_CTX–M–14_ (followed by *bla*_CTX–M–27)_ as the dominant ESBL genes in the GMS similar to other regions in the world (Bevan et al., 2017). Genotypes *bla*_CTX–M–15_ and *bla*_CTX–M–14_ have successfully displaced other ESBL genes (*bla*_CTX–M–1_ and others_)_ perhaps owing to their stable maintenance in commensal Ec within humans and animal gastro-intenstinal tract and have resulted in a consistently upward carriage trend all over the world including GMS (Karanika et al., 2016). Carbapenemase has also been increasingly detected from both clinical and carriage specimens with *bla*_NDM_ being the most commonly detected genotype, followed by *bla*_OXA_ The major carbapenemase found in GMS were *bla*_NDM_ genes similar to South Asia and other SEA nations (Hsu et al., 2017). The other most commonly reported carbapenemase was *bla*_OXA–48_ like genes followed by *bla*_IMP,_ *bla*_VIM_ and *bla*_KPC._ It is interesting to note that *bla*_KPC_ were detected in both Ec and Kp from Vietnam and Thailand hinting at transfer of KPC mechanisms via mobile genetic elements from Kp to Ec (Ruppé et al., 2015).

Our study also found that ESBL isolation in both hospital as well as community settings is significantly associated with recent exposure to broad-spectrum antibiotics such as third-generation cephalosporin and fluoroquinolone similar to the risk factors reported by other recent systematic review and meta-analysis in carriage and infection studies (Karanika et al., 2016; Flokas et al., 2017; Larramendy et al., 2020). Another factor-chronic kidney disease is frequently associated with ESBL-E carriage, however, the disease has not been proven as a causative factor for ESBL-E carriage and further research is needed to explain this association (Kiddee et al., 2019). However, patients with chronic kidney disease often have history of frequent hospitalization and invasive procedures which have been the most common reported factors associated with ESBL-E infection (Flokas et al., 2017; Larramendy et al., 2020). Interestingly, western studies also reported international travel as one of the risk factors associated with ESBL isolation. AMR has multifactorial drivers and hence it is crucial to identify other risk factors from a one-health perspective that may be causing drug resistance (Chereau et al., 2017). Antibiotic use is largely unregulated in the rapidly developing and populated GMS (Klein et al., 2018), which may be one of the driving factors of increasing ESBL prevalence. We did not include antibiotic consumption patterns of the GMS nations in this review, nevertheless, correlating antibiotic consumption patterns with prevalence of ESBL-E and CPE is an important question warranting further research. Moreover, it is unwise to discount the environmental factors associated with a higher risk of ESBL spread, such as farming and household practices, as reported in Thai, Vietnamese and Cambodian studies from rural regions (Niumsup et al., 2018; Atterby et al., 2019; Trung et al., 2019; van Aartsen et al., 2019). A review study by Collignon et al. (2018) has reported that environmental and socioeconomic factors such as higher temperatures, ratio of private to public health expenditure and poor governance were positively correlated with AMR. GMS shares a common geography and social background, and thus, it is vital to have collaborative studies across the region that can produce a homogenous data set for assessment and comparison of ESBL and carbapenemase from the region. Also, Thailand and Vietnam serve as global hubs for recreational and medical tourism, and have been identified as possible exporters of novel ESBL and carbapenemase genes with the patients and travelers that visit the GMS (Tham et al., 2010; Barreto Miranda et al., 2016; Nakayama et al., 2018). It is interesting to note that few regional studies such as Study for Monitoring Antimicrobial Resistance Trends (SMART) with a standard methodology are a step in the right direction for comparison of data across nations (Hawser et al., 2009; Huang et al., 2012; Chang et al., 2017; Jean et al., 2018). Furthermore, uptake of software application such as AMASS (Antimicrobial resistance Surveillance system) that can analyze routine hospital microbiology laboratory data for generation of standardized surveillance reports will be useful in monitoring AMR in local settings (Lim et al., 2020). Upcoming projects of ACORN (A Clinically Oriented Antimicrobial Resistance Surveillance Network) study funded by Wellcome grant that focus on capturing patient outcomes and linking them with microbiology data within the current available resources seem promising and may help in informing treatment guidelines and providing comparable data for monitoring AMR burden and effectiveness of interventions (van Doorn et al., 2020).

This review study was focused on ESBL and carbapenemase enzymes and their phenotypic and genotypic detection. Hence, we have excluded studies that reported phenotypic 3GC or carbapenem resistance using antibiotic susceptibility tests, more commonly reported in provincial hospital, and thus missed these datasets from our review. There is considerable overlap between ESBL and carbapenemase-mediated resistance with phenotypic resistance (van Hoek et al., 2011). Thus, the stark gaps in ESBL and carbapenemase data availability cannot be extrapolated to absence of phenotypic resistance data, especially of third generation cephalosporins and carbapenems which have been published in previous systematic reviews conducted for SEA countries (Suwantarat and Carroll, 2016; Malchione et al., 2019). However, with the focus on GMS, the unavailability of data becomes more evident with even fewer studies in Laos, Myanmar and Yunnan and Guangxi provinces of China. Another limitation of this study is that we included published data including surveillance reports and cross-sectional studies which may not be consistent and inclusive of the internal nationwide surveillance reports mandated by individual countries in the GMS. GMS nations such as Laos and Vietnam do not grant public access to their surveillance data whereas the National Antimicrobial Resistance Surveillance Centre, Thailand (NARST) has been publishing surveillance data biannually for universal access (Chua et al., 2021). However, since our study did not capture any data points from NARST and it does not provide the exact surveillance information which can be accessed on NARST portal.^2^ Also, we did not actively search for official websites for Chinese surveillance data.

Poor antibiotic regulation and environmental factors have led to an unprecedented rise in ESBL, specifically ESBL-Ec isolation from clinical and carriage specimens. With rising resistance to beta-lactams, third-generation cephalosporins and fluoroquinolones, there will be increased demand for carbapenems, one of the last-line antibiotics, which may eventually drive an increase in CPE genes as has already been detected in specimens across the GMS. Thus, investing in collaborative research studies with standardization in diagnostic methods for detection of ESBL, carbapenemase and other antibiotic susceptibility testings exploring the risk factors associated with ESBL and carbapenemase isolation in hospital as well as community as well as strict surveillance and enforcement of infection-prevention and control measures, antibiotic regulations, and effective treatment strategies are needed to limit the rising prevalence of ESBL and Carbapenemase in GMS.

## Author Contributions

LH, SS, and AT contributed to conception and design of the study. SS and AT performed the database search, subsequent screening of articles for inclusion in the review, and extracted data from included articles for data analysis. AT, KP, and SS performed the statistical analysis and prepared the figures included in the systematic review. SS wrote the first draft of the manuscript. All authors contributed to manuscript revision, read, and approved the submitted version.

## Conflict of Interest

The authors declare that the research was conducted in the absence of any commercial or financial relationships that could be construed as a potential conflict of interest.

## Publisher’s Note

All claims expressed in this article are solely those of the authors and do not necessarily represent those of their affiliated organizations, or those of the publisher, the editors and the reviewers. Any product that may be evaluated in this article, or claim that may be made by its manufacturer, is not guaranteed or endorsed by the publisher.

## Footnotes

1 About the Greater Mekong Subregion. https://www.greatermekong.org/about (Accessed March 10, 2021).

2 NARST, 2021. National Antimicrobial Resistant Surveillance Center, Thailand. http://narst.dmsc.moph.go.th/ (Accessed March 15, 2021).
