# Supplementary material for: Epidemiology of Extended-Spectrum Beta-Lactamase and Carbapenemase-Producing Enterobacterales in the Greater Mekong Subregion: A Systematic-Review and Meta-Analysis of Risk Factors Associated With Extended-Spectrum Beta-Lactamase and Carbapenemase Isolation
Source: Front Microbiol. 2021 Nov 26;12:695027. doi: 10.3389/fmicb.2021.695027 (PMC8661499; doi:10.3389/fmicb.2021.695027)
Supplement: Supplementary file 1 [file Data_Sheet_1.docx]

**Supplementary material**

**Table S1: ESBL and carbapenemase prevalence (proportion) in *E. coli* in GMS**

| **Year of study** | | **Setting** | **Place** | **Site of isolates** | **Sample size** | **ESBL positive (%)** | **CPE positive(%)** | **Reference (ID)** |
| --- | --- | --- | --- | --- | --- | --- | --- | --- |
| **Thailand** | | | | | | | | |
| 2004 | | 1 tertiary hospital | Songkla | Blood | 59 | 5.1% | Not tested | (Jitsurong and Yodsawat, 2006) |
| 2005-2006 | | 1 tertiary hospital | Khon Kaen | Blood | 145 | 22% | Not tested | (Anunnatsiri et al., 2012) |
| 2007-2008 | | 1 tertiary hospital | Bangkok | Blood | 97 | 21.6% | Not tested | (Musikatavorn et al., 2011) |
| 2008-2014 | | Hospital (2 provincial, 2 military and 16 district) | Sa kaeo; Nakhon Phanom | Blood | 3293 | Range from 19.6% (2008) to 32.7% (2013);  CAI: 27%  HAI: 51% | 0.12% (2014)  *bla*_NDM-1_ (n=2 in ESBL +ve)  *bla*_IMP_ (n =2) in ESBL -ve | (Rhodes et al., 2019; Sawatwong et al., 2019) |
| 2010-2013 | | 1 tertiary hospital | Bangkok | Urine | Not given | 2010: 36.8%  2011: 35.6%  2012: 54.2%  2013: 33.3% | Not tested | (Jean et al., 2016) |
| 2013-2014 | | Clinics | Tak | Urine | 67 | 20.9% | Not tested | (Chalmers et al., 2015) |
| 2013 | | 3 hospitals | Phuket | Urine | 113 | 46% | Not tested | (Themphachana et al., 2014) |
| 2014 | | 1 tertiary hospital | Nakhon Si Thammarat | Urine | 254 | 50% | Not tested | (Themphachanal et al., 2015) |
| 2007 | | 2 centres | Not given | Intra-abdominal | 65 | 50.8% | Not tested | (Hawser et al., 2009) |
| 2002-2010 | | 2 tertiary hospital | Bangkok, Songkla | Intra-abdominal | - | 43.1% | Not tested | (Huang et al., 2012) |
| 2010-2013 | | 2 tertiary hospital | Bangkok, Songkla | Intra-abdominal | - | 49.8% | Not tested | (Chang et al., 2017) |
| 2001-2010 | | Diarrhoea patients | Not given | Faecal | 213 | 0.5% | Not tested | (Nirdnoy et al., 2017) |
| 2007-2008 | | Diarrhoea patients | Not given | Faecal | 336 | 22% | Not tested | (Tham et al., 2010) |
| 2000-2005 | | 1 tertiary hospital | Bangkok | All (HAI) | 2000-02: 1863  2003-05: 1884 | 2000-02: 0.9%  2003-05: 19.9% | Not tested | (Tantracheewathorn et al., 2007) |
| 2002 | | 1 tertiary hospital | Bangkok | All clinical isolates | 212 | 17% | Not tested | (Udomsantisuk et al., 2011) |
| 2000-2005 | | 28 hospitals | All across Thailand | All clinical isolates | 2000: 24,918  2001: 47,020  2002: 44,261  2003: 44,416  2004: 55,479  2005: 41,734 | 2000: 18.7%  2001: 51.6%  2002: 54.1%  2003: 54.4%  2004: 52.1%  2005: 29.1% | Not tested | (Polwichai et al., 2011) |
| 2003-2005 | | 1 tertiary hospital | Songkla | All clinical isolates | 355 | 12.6% | Not tested | (Phongpaichit et al., 2011) |
| 2004 | | 1 tertiary hospital | Bangkok | All clinical isolates | 120 | 31% | Not tested | (Pongpech et al., 2008) |
| 2004-2005 | | 1 tertiary hospital | Bangkok | All clinical isolates | 1776 | 13.2% | Not tested | (Kiratisin et al., 2008a) |
| 2004-2010 | | 10 provincial hospitals | Northeast Thailand | All clinical isolates | 3525 | 2004: 2.92%;  2010: 18% | Not tested | (Kanoksil et al., 2013) |
| 2005-2006 | | 1 tertiary hospital | Bangkok | All clinical isolates | 1959 | 33.5% | Not tested | (Chaiwarith et al., 2008) |
| 2006 | | 1 tertiary hospital | Prathumthani | All clinical isolates | 200 | 26% | Not tested | (Apisarnthanarak et al., 2008) |
| 2005-2007 | | 1 tertiary hospital | Bangkok | All clinical isolates | 10,353 | 34.7% | Not tested | (Kiratisin et al., 2008b) |
| 2012-2013 | | 1 tertiary hospital | Bangkok | All clinical isolates | 375 | 33% | Not tested | (Sethaphanich et al., 2016) |
| 2015-2017 | | 3 centres | Not given | All clinical isolates | 357 | 51.8% | 3% | (Wen-Chien and Stone, 2020) |
| **Vietnam** | | | | | | | | |
| 2011-2013 | 1 tertiary hospital | | Hanoi | Blood | 131 | 45% | Not tested | (Dat et al., 2017) |
| 2011-2013 | 1 tertiary hospital | | Ho Chi Minh | Blood | 445 | 36.4% | Not tested | (Lan et al., 2017) |
| 2012-2014 | 2 tertiary hospitals | | Hanoi | Blood | 56 | 39.3% | Not tested | (Hung et al., 2019) |
| 2010-2011 | 4 tertiary hospitals | | Hanoi;  Ho Chi Minh | Urine | 225 | 62.7% | Not tested | (Thi Quynh Nhi et al., 2018) |
| 2013 | 1 hospital | | Nha Trang | Urine | 51 | 45.1% | Not tested | (Hoang et al., 2017b) |
| 2002-2010 | 3 tertiary hospitals | | Hanoi;  Binh Dan;  Cho Ray | Intra-abdominal | - | 38.6% | Not tested | (Huang et al., 2012) |
| 2007 | 2 centres | | Not given | Intra-abdominal | 122 | 34.4% | Not tested | (Hawser et al., 2009) |
| 2009-2011 | 4 tertiary hospitals | | Hanoi;  Ho Chi Minh | Intra-abdominal | 697 | 48.1% | 0% | (Biedenbach et al., 2014) |
| 2010-2013 | 4 centres | | Hanoi;  Ho Chi Minh | Intra-abdominal | - | 47.9% | Not tested | (Chang et al., 2017) |
| 2004 | 1 tertiary hospital | | Ho Chi Minh | All clinical isolates | 150 | 19.3% | Not tested | (Jones et al., 2006) |
| **Cambodia** | | | | | | | | |
| 2007-2010 | | 1 hospital | Phnom Penh | Blood | 130 | 47.7% | Not tested | (Vlieghe et al., 2013) |
| 2004-2005 | | 1 reference laboratory | Phnom Penh | Urine | 93 | 36.6% | Not tested | (Ruppé et al., 2009) |
| 2007-2011 | | 1 hospital | Siem Reap | Urine | 170 | 36.4% | Not tested | (Moore et al., 2016) |
| 2012-2015 | | 1 reference laboratory | Phnom Penh | All clinical isolates | 789 | 42.7% | Not tested | (Caron et al., 2018) |
| **Myanmar** | | | | | | | | |
| 2016 | | 1 tertiary hospital | Yangon | All clinical isolates | 426 | 36.9% | 8.2% | (Aung et al., 2018) |
| 2019 | | 1 tertiary hospital | Yangon | All clinical isolates | 123 | 76.4% | Not tested | (San et al., 2021) |
| **Yunnan and Guangxi province of China** | | | | | | | | |
| 2009-2010 | | 3 hospitals | Dali Bai | All clinical isolates | 180 | 48.4% | Not tested | (Zhao et al., 2014) |

**Table S2: ESBL and carbapenemase prevalence (proportion) in *K. pneumoniae* in GMS**

| **Year of study** | **Setting** | **Place** | **Site of isolates** | **Sample size** | **ESBL positive (%)** | **CPE positive(%)** | **Reference (ID)** |
| --- | --- | --- | --- | --- | --- | --- | --- |
| **Thailand** | | | | | | | |
| 2004 | 1 tertiary hospital | Songkla | Blood | 36 | 44.4% | Not tested | (Jitsurong and Yodsawat, 2006) |
| 2007-2008 | 1 tertiary hospital | Bangkok | Blood | 38 | 23.7% | Not tested | (Musikatavorn et al., 2011) |
| 2008-2014 | Hospital (2 provincial, 2 military and 16 district) | Sa kaeo; Nakhon Phanom | Blood | 1059 | 27.4%  CAI: 23%  HAI: 55% | 0.37%  (*bla*_NDM-1_) | (Rhodes et al., 2019; Sawatwong et al., 2019) |
| 2010-2013 | 1 tertiary hospital | Bangkok | Urine | - | 2010: 40%  2011: 71.4%  2012: 53.9%  2013: 50% | Not tested | (Jean et al., 2016) |
| 2013 | 3 hospitals | Phuket | Urine | 52 | 57% | Not tested | (Themphachana et al., 2014) |
| 2007-2009 | 1 hospital | Bangkok | Tracheal aspirate | 44 | 65.9% | Not tested | (Werarak et al., 2010) |
| 2008-2009 | 1 hospital | Khon Kaen | Tracheal aspirate | - | 47% | Not tested | (Reechaipichitkul et al., 2013) |
| 2007 | 2 centres | Not given | Intra-abdominal | 33 | 45.5% | Not tested | (Hawser et al., 2009) |
| 2002-2010 | 2 tertiary hospital | Bangkok;  Songkla | Intra-abdominal | - | 40.7% | Not tested | (Huang et al., 2012) |
| 2010-2013 | 2 tertiary hospitals | Bangkok;  Songkla | Intra-abdominal | - | 36.5% | Not tested | (Chang et al., 2017) |
| 2000-2001 | 1 tertiary hospital | Bangkok | All clinical isolates | 400 | 26% | Not tested | (Kusum et al., 2004) |
| 2000-2002 | 1 tertiary hospital | Bangkok | All clinical isolates from HAI | 1863 | 1% | Not tested | (Tantracheewathorn et al., 2007) |
| 2000-2005 | 28 hospitals | All across Thailand | All clinical isolates | 2000: 15,486  2001: 18,709  2002: 17,337  2003: 20,323  2004: 23,108  2005: 25,459 | 2000: 34.0%  2001: 36.9%  2002: 35.9%  2003: 37.2%  2004: 39.7%  2005: 39.7% | Not tested | (Polwichai et al., 2011) |
| 2002 | 1 tertiary hospital | Bangkok | All clinical isolates | 54 | 34.5% | Not tested | (Udomsantisuk et al., 2011) |
| 2003-2005 | 1 tertiary hospital | Bangkok | All clinical isolates from HAI | 1884 | 29.5% | Not tested | (Tantracheewathorn et al., 2007) |
| 2004-2010 | 10 provincial hospitals | Northeast Thailand | All clinical isolates | 1032 | 2004:10%  2010:16.4% | Not tested | (Kanoksil et al., 2013) |
| 2004-2005 | 1 tertiary hospital | Bangkok | All clinical isolates | 1001 | 12.7% | Not tested | (Kiratisin et al., 2008a) |
| 2005-2006 | 1 tertiary hospital | Bangkok | All clinical isolates | 1185 | 57% | Not tested | (Chaiwarith et al., 2008) |
| 2006 | 1 tertiary hospital | Prathumthani | All clinical isolates | 96 | 21% | Not tested | (Apisarnthanarak et al., 2008) |
| 2005-2007 | 1 tertiary hospital | Bangkok | All clinical isolates | 5483 | 33.6% | Not tested | (Kiratisin et al., 2008b) |
| 2012-2013 | 1 tertiary hospital | Bangkok | All clinical isolates | 190 | 41% | Not tested | (Sethaphanich et al., 2016) |
| 2015-2017 | Not given | Not given | All clinical isolates | 358 | 35.2% | 9.8% | (Wen-Chien and Stone, 2020) |
| **Vietnam** | | | | | | | |
| 2011-2013 | 1 tertiary hospital | Hanoi | Blood | 133 | 12.3% | Not tested | (Dat et al., 2017) |
| 2002-2010 | 3 tertiary hospitals | Hanoi;  Binh Dan;  Cho Ray | Intra-abdominal |  | 28.9% | Not tested | (Huang et al., 2012) |
| 2009-2011 | 4 tertiary hospitals | Hanoi;  Ho Chi Minh | Intra-abdominal | 129 | 39.5% | 2.3%  (*bla*_NDM-1_, *bla*_KPC-2_, *bla*_OXA-48_, *bla*_VIM_) | (Biedenbach et al., 2014) |
| 2010-2013 | 4 centres | Hanoi;  Ho Chi Minh | Intra-abdominal |  | 30.4% | Not tested | (Chang et al., 2017) |
| **Cambodia** | | | | | | | |
| 2007-2010 | 1 hospital | Phnom Penh | Blood | 32 | 43.8% | Not tested | (Vlieghe et al., 2013) |
| 2007-2009 | 2 hospitals | Not given | Blood, sputum, nasopharyngeal swabs | 47 | 17% | Not tested | (Rammaert et al., 2012) |
| 2012-2015 | 1 reference laboratory | Phnom Penh | All clinical isolates | 243 | 33.7% | Not tested | (Caron et al., 2018) |
| **Myanmar** | | | | | | | |
| 2018 | 1 tertiary hospital | Yangon | All clinical isolates | 191 | 33.5% | 7.3% | (Aung et al., 2021) |

**Table S3: ESBL and carbapenemase prevalence (proportion) in *Enterobacterales* in GMS**

| **Year of study** | **Setting** | **Place** | **Site of isolates** | **Sample size** | **ESBL positive(%)** | **CPE positive(%)** | **Reference** |
| --- | --- | --- | --- | --- | --- | --- | --- |
| **Thailand** | | | | | | | |
| 2010 | 10 Clinical centres | Not given | All clinical isolates | 239 | 45.2% | 0.4% | (Kiratisin et al., 2012) |
| 2010-2017 | 1 tertiary hospital | Bangkok | Blood | 97 | 53.2% | Not tested | (Nivesvivat et al., 2018) |
| 2014 | 1 tertiary hospital | Prachuab Khiri Khan Province | All clinical isolates | 624 | - | 1.1%  (*bla*_NDM_) | (Preechachuawong et al., 2015) |
| **Vietnam** | | | | | | | |
| 2010 | 3 centres | Not given | All clinical isolates | 71 | 55.1% | Not tested | (Kiratisin et al., 2012) |
| 2010-2012 | 1 surgical hospital | Hanoi | All clinical isolates | 4096 | - | 1.7%  (*bla*_NDM-1_ (68.1%)) followed by (*bla*_MBL_, (*bla*_OXA-48_, (*bla*_KPC_) | (Tran et al., 2015) |
| **Cambodia** | | | | | | | |
| 2007-2010 | 1 hospital | Phnom Penh | Blood | 183 | 48.6% | Not tested | (Vlieghe et al., 2015) |
| **Myanmar** | | | | | | | |
| 2014 | 3 hospitals | Yangon | Blood | 42 | 38% | 14% | (Myat et al., 2017) |
| 2015 – 2016 | 1 tertiary hospital | Yangon | Blood | 70 | 25.7 | Not tested | (Myat et al., 2020) |
| **Laos** | | | | | | | |
| 2010-2014 | 1 tertiary hospital | Vientiane | Blood | 360 | 20% | Not tested | (Chang et al., 2020) |

**Table S4: Carriage ESBL E prevalence proportion**

| **Year** | **Place** | **Setting (inclusion criteria)** | **Age group** | **Sample size** | **ESBL Prevalence %** | **CPE prevalence %** | **Reference** |
| --- | --- | --- | --- | --- | --- | --- | --- |
| **Thailand** | | | | | | | |
| 2008 | Kanchanaburi | community (no Abx tt in prior 3 months) | adults | 141 | 61.7 | Not tested | Sasaki et al., 2010 |
| 2009 | Nan | community (no Abx tt in prior 3 months) | adults | 147 | 32 | Not tested | Luvsansharav et al., 2011 |
| 2009 | Nakhon Si Thammarat | community (no Abx tt in prior 3 months) | adults | 144 | 32.6 | Not tested | Luvsansharav et al., 2011 |
| 2009 | Kanchanaburi | community (no Abx tt in prior 3 months) | adults | 154 | 53.6 | Not tested | Luvsansharav et al., 2011 |
| 2009-10 | Bangkok | hospital (suspect prostate carcinoma) | adults | 144 | 25.7 | Not tested | Siriboon et al., 2012 |
| 2010 | Kanchanaburi | community (healthy individuals) | adults | 417 | 69.3 | Not tested | Luvsansharav et al., 2012 |
| 2012-13 | central province | food factory, farm workers (healthy) | adults | 544 | 75.5 | Not tested | Boonyasiri et al., 2014 |
| 2013 | Wang Thong | community (healthy individuals) | adults | 307 | 58.6 | 0 | Niumsup et al., 2018 |
| 2014-15 | Phitsanulok | hospital (ICU patients with no GIT illness) | adults | 215 | 62.3 | 0 | Kiddee et al., 2019 |
| **Cambodia** | | | | | | | |
| 2011 | Kampong Cham | Community | all age groups | 307 |  | 1 | Atterby et al., 2018 |
| **Laos** | | | | | | | |
| 2011 | Vientiane | Community (child care) | children | 397 | 23.2 | 0 | Stoessor et al., 2015 |

**Table S5: Carriage of ESBL and Carbapenemase in *E. coli***

| **Year** | **Place** | **Setting (inclusion criteria)** | **Age group** | **Sample size** | **ESBL Prevalence%** | **CPE prevalence %** | **Reference** |
| --- | --- | --- | --- | --- | --- | --- | --- |
| **Thailand** | | | | | | | |
| 2004 | Bangkok | Healthy adults | adults | 120 | 0 | Not tested | Pongpech et al., 2008 |
| 2011 | Bangkok | Hospital (patients attending ER) | adults | 452 | 16 | Not tested | Pornsinchai et al., 2015 |
| 2013 | Amphor Mueang | Community (healthy adults) | adults | 223 | 49.8 | 0 | Seenama et al., 2019 |
| 2013-14 | Bangkok | • hospitalized in general medical wards for less than 24h (past history)  • Within 48h after hospitalization (current) | adults | 487 | 42.3 | Not tested | Rattanaumpawan et al., 2018 |
| 2014-15 | Phitsanulok | Adult patients (ICU) above 20 years and without intestinal tract infections | adults | 160 | 67.5 | 0 | Kiddee et al., 2019 |
| 2014-15 | Lamphun; Chonburi | Community (healthy individuals) | adults | 534 | 66.5 | Not tested | Khamsarn et al., 2016 |
| 2015-16 | Chiang Mai;  Lamphun | Community (Healthy farm workers) | adults | 113 | 50.4 | Not tested | Nuangmek et al., 2018 |
| 2015-16 | Chonburi | Community (Healthy farm workers) | adults | 83 | 60.2 | Not tested | Nuangmek et al., 2018 |
| **Vietnam** | | | | | | | |
| 2012-13 | Tien Giang | Community (farmers) | adults | 204 | 32 | 0 | Nguyen et al., 2019 |
| 2012-13 | Tien Giang | Community (rural, adults) | adults | 204 | 49.5 | 0 | Nguyen et al., 2019 |
| 2012-13 | Tien Giang | Community (urban, adults) | adults | 102 | 38.2 | 0 | Nguyen et al., 2019 |
| 2013 | Ho Chi Minh | Community (healthy adults) | adults | 103 | 9.7 | Not tested | Hoang et al., 2017 |
| 2013 | Nha Trang | Community | adults | 83 | 73.5 | Not tested | Hoang et al., 2017 |
| 2013-14 | Bavi province | Commmunity (healthy individuals) | adults | 51 | 54.9 | Not tested | Bui et al., 2018 |
| 2013-16 | Thai Binh | Community (Healthy farm workers) | adults | 451 | 83.1 | Not tested | Kawahara et al., 2019 |
| 2014-16 | Ho Chi Minh | • readmission to hospital within 90 days • within 48 hours of ICU admission | adults | 740 | 59.1 | 1.1 | Thuy et al., 2017 |
| 2015 | Kien Xuong;  Soc Son | Community (Pig farm workers) | adults | 163 | 71.8 | Not tested | Dang et al., 2018 |
| 2016 | Ho Chi Minh | Hospital (healthy children) | children | 498 | 83.7 | Not tested | Thi et al., 2018 |
| **Cambodia** | | | | | | | |
| 2019 | Siem Reap | Community | all age groups | 290 | 92.8 | 1.4 | Singh et al., 2020 |

**Table S6: Carriage of ESBL and Carbapenemase in *K. pneumoniae***

| **Year** | **Place** | **Setting (inclusion criteria)** | **Age group** | **Sample size** | **ESBL Prevalence %** | **CPE prevalence %** | **Reference** |
| --- | --- | --- | --- | --- | --- | --- | --- |
| **Thailand** | | | | | | | |
| 2011 | Bangkok | patients attending ER | adults | 452 | 1 | Not tested | Pornsinchai et al., 2015 |
| 2013-14 | Bangkok | • hospitalized in general medical wards for less than 24h (past history)  • Within 48h after hospitalization (current) | adults | 487 | 16.6 | Not tested | Rattanaumpawan et al., 2018 |
| 2014-15 | Phitsanulok | Adult patients (ICU) above 20 years and without intestinal tract infections | adults | 160 | 19.4 | 0 | Kiddee et al., 2019 |
| **Cambodia** | | | | | | | |
| 2015-16 | Not given | Pregnant women (samples taken at delivery or shortly after delivery in the case of home delivery) | adults | 146 | 14.4 | Not tested | Huynh et al., 2020 |
| 2019 | Siem Reap | Community | all age groups | 290 | 44.1 | 0.7 | Singh et al., 2020 |

**Table S7: Identification of ESBL- *E. coli* genes from clinical and non-clinical isolates in GMS**

| **Genes** | | **ESBL- *E. coli*** | | | | **CPE** |
| --- | --- | --- | --- | --- | --- | --- |
| GMS nations (ref) | | *bla*_CTX-M_ sub-types | *bla*_TEM_ | *bla*_SHV_ | Others |  |
| Cambodia | Clinical  (Ruppé et al., 2009; Hout et al., 2015; Stoesser et al., 2015a; Nadimpalli et al., 2019) | -55, -14, -27, -15 | -1 |  | *bla*_OXA-1_ | *bla*_NDM-5,_ *bla*_OXA-181,_ |
|  | Carriage  (Atterby et al., 2019; Nadimpalli et al., 2019) | -1, -9, -55, -15, |  |  |  | *bla*_OXA-48,_ |
| China | Clinical  (Zhao et al., 2014) | Group 1, group 9 |  |  |  |  |
| Laos | Clinical (Cusack et al., 2019) |  |  |  |  | *bla*_NDM_ |
|  | Carriage (Stoesser et al., 2015b) | -14, -55, -15, -27, -64, -24, -101 |  | -2 |  |  |
| Myanmar | Clinical (Myat et al., 2017; Sugawara et al., 2017; Aung et al., 2018; San et al., 2021) | -15, -14, -27, -55, | *bla*_TEM_ | -2 |  | *bla*_NDM-5,-4, -7, -1,_ *bla*_OXA-181_ |
| Thailand | Clinical (Girlich et al., 2001; Apisarnthanarak et al., 2007; Apisarnthanarak et al., 2008; Kiratisin et al., 2008a; Niumsup et al., 2008; Pongpech et al., 2008; Udomsantisuk et al., 2011; Rimrang et al., 2012; Lunha et al., 2015; Netikul and Kiratisin, 2015; Pornsinchai et al., 2015; Preechachuawong et al., 2015; Kazmierczak et al., 2016; Paveenkittiporn et al., 2016; Stoesser et al., 2016; Cha et al., 2017; Matsumura et al., 2017; Bubpamala et al., 2018; Kerdsin et al., 2019; Sawatwong et al., 2019) | -15, -55, -9, -14, -27, -40 | -1A, -1B | -4, -5, -12, -27, -28, -4, -11, -28, -12, -13 | *bla*_OXA-10,_  *bla*_VEB-1,_ *bla*_PER,_ *bla*_GES_ | *bla*_NDM-1,_ *bla*_OXA-48-like (-48, -181, 232)_  *bla*_IMP-14,_ *bla*_KPC-13,_ *bla*_KPC-2_ |
|  | Carriage (Pornsinchai et al., 2015; Seenama et al., 2019; Sukkua et al., 2019) | -15, -14, -161 | -1B, -116 | -12, -148 |  |  |
| Vietnam | Clinical  (Cao et al., 2002; Breurec et al., 2013; Hoang et al., 2013; Trang et al., 2013; Biedenbach et al., 2014; Jakobsen et al., 2014; Jean et al., 2017; Lan et al., 2017; Hoang et al., 2019; Honda et al., 2019; Hung et al., 2019; L et al., 2019) | -15, -73, -98, -161, -24, -27, -55 | -1, -79, -82 | -5, -7, -12,-15, -24, -33, -57, -77 | *bla*_OXA-4,_ *bla*_VEB-1_ | *bla*_NDM-4, -5_, *bla*_NDM-1,_ *bla*_KPC-2,_ |
|  | Carriage (Hoang et al., 2017a; Bui et al., 2018; Thi Quynh Nhi et al., 2018; Trung et al., 2019) | -15, -55, -11, -14, -27, -65, -24, -3, -64 | *bla*_TEM_ | *bla*_SHV_ | *bla*_OXA,_ *bla*_VEB-1,_ *bla*_OXA-10, -160, -40, -4_ |  |

**Table S8: Identification of ESBL- *K. pneumoniae* genes from clinical and carriage isolates in GMS**

| Genes | | ESBL- *K. pneumoniae* | | | | CPE |
| --- | --- | --- | --- | --- | --- | --- |
| GMS nations (ref) | | *bla*_CTX-M_ sub-types | *bla*_TEM_ | *bla*_SHV_ | Others |  |
| Cambodia | Clinical (Ruppé et al., 2009) | *bla*_CTX-M_ | *bla*_TEM_ | *bla*_SHV_ |  |  |
|  | Carriage (Atterby et al., 2019) | -27, -14, -15, |  | -2, -11, 28 |  | *bla*_OXA-48_ |
| China | Clinical (Zheng et al., 2016) | -15 | -1 | -1 |  | *bla*_NDM-1,_ *bla*_IMP-4_ |
| Laos | Carriage (Stoesser et al., 2015b) | -14 |  | -2a |  |  |
| Myanmar | Clinical (Aung et al., 2021) | Group -1, group -9 |  | -27 |  | *bla*_NDM-5, -1, -7_ |
| Thailand | Clinical (Chanawong et al., 2001; Girlich et al., 2001; Chanawong et al., 2007; Kiratisin et al., 2007; Apisarnthanarak et al., 2008; Kiratisin et al., 2008a; Niumsup et al., 2008; Udomsantisuk et al., 2011; Rimrang et al., 2012; Netikul et al., 2014; Lunha et al., 2015; Netikul and Kiratisin, 2015; Kazmierczak et al., 2016; Paveenkittiporn et al., 2016; Matsumura et al., 2017; Runcharoen et al., 2017a; Runcharoen et al., 2017b; Srijan et al., 2018; Kerdsin et al., 2019; Sawatwong et al., 2019) | -15, -55, -14, -3, -27, -9, | -1, -1A, -1**B,** | -12, -1, -2a, -11, -27, -28, -71, -75, -30 | *bla*_VEB-1,_ *bla*_OXA-10, -9, -2_ | *bla*_NDM-1,_ *bla*_OXA-48-like (-48, -181, 232)_  *bla*_IMP-14a,_ *bla*_KPC-13,_ *bla*_KPC-2_ |
|  | Carriage (Pornsinchai et al., 2015; Kiddee et al., 2018) | -15, -161 | -1, -116 | -12, -148 |  | *bla*_NDM_ |
| Vietnam | Clinical (Cao et al., 2002; Hoang et al., 2013; Biedenbach et al., 2014; Jean et al., 2017; Tada et al., 2017; Berglund et al., 2018) | -15, -55, -14, -24, -27 | -1, -1B, -199 | -12, -29, -11, -12, -28, -55, -5, -1, -73, -155 | *bla*_OXA-9_ | *bla*_KPC-2,_ *bla*_NDM-1,_ *bla*_NDM-4,_ *bla*_OXA-48,_ *bla*_VIM_ |

**Table S9: Identification of ESBL genes from Enterobacterales from clinical and carriage isolates in GMS**

| Genes | | ESBL- *Enterobacterales* | | | | CPE |
| --- | --- | --- | --- | --- | --- | --- |
| GMS nations (ref) | | *bla*_CTX-M_ sub-types | *bla*_TEM_ | *bla*_SHV_ | Others |  |
| Cambodia | Carriage (van Aartsen et al., 2019) | 15, -55, -14, -24, -27 |  |  |  |  |
| Myanmar | Clinical (Myat et al., 2017) | -15 |  |  |  | *bla*_NDM-4, -7, -5, -1._ *bla*_OXA-181_ |
| Thailand | Clinical (Sheng et al., 2013; Barreto Miranda et al., 2016; Jean et al., 2017; Jean et al., 2018; Laolerd et al., 2018) | -15, -55, -9, -1, 14, -27, -65, -63 | -1A, -1B | -12, | *bla*_OXA-10_ | *bla*_NDM-1_, *bla*_OXA-48,_ *bla*_KPC_ *,bla*_IMP-14_ |
|  | Carriage (Sasaki et al., 2010; Luvsansharav et al., 2011; Niumsup et al., 2018; Kiddee et al., 2019) | -15, -55, -14, -27, -65, -63, -161, (group IV, III, I) |  | - |  | *bla*_NDM-1_ |
| Vietnam | Clinical (Sheng et al., 2013; Jean et al., 2017) | -15 |  |  |  |  |

# **Table S10: PRISMA 2020 Main Checklist**

| **Topic** | **No.** | **Item** | **Location where item is reported** |
| --- | --- | --- | --- |
| **TITLE** |  |  |  |
| **Title** | 1 | Identify the report as a systematic review. | Title, page 1 |
| **ABSTRACT** |  |  |  |
| **Abstract** | 2 | See the PRISMA 2020 for Abstracts checklist |  |
| **INTRODUCTION** |  |  |  |
| **Rationale** | 3 | Describe the rationale for the review in the context of existing knowledge. | Line 105-109 |
| **Objectives** | 4 | Provide an explicit statement of the objective(s) or question(s) the review addresses. | Line 109-113 |
| **METHODS** |  |  |  |
| **Eligibility criteria** | 5 | Specify the inclusion and exclusion criteria for the review and how studies were grouped for the syntheses. | Line 122-142 |
| **Information sources** | 6 | Specify all databases, registers, websites, organisations, reference lists and other sources searched or consulted to identify studies. Specify the date when each source was last searched or consulted. | Line 114-121 |
| **Search strategy** | 7 | Present the full search strategies for all databases, registers and websites, including any filters and limits used. | Line 116-121 |
| **Selection process** | 8 | Specify the methods used to decide whether a study met the inclusion criteria of the review, including how many reviewers screened each record and each report retrieved, whether they worked independently, and if applicable, details of automation tools used in the process. | Line 116-142 |
| **Data collection process** | 9 | Specify the methods used to collect data from reports, including how many reviewers collected data from each report, whether they worked independently, any processes for obtaining or confirming data from study investigators, and if applicable, details of automation tools used in the process. | Line 144-152 |
| **Data items** | 10a | List and define all outcomes for which data were sought. Specify whether all results that were compatible with each outcome domain in each study were sought (e.g. for all measures, time points, analyses), and if not, the methods used to decide which results to collect. | Line 122-135 |
|  | 10b | List and define all other variables for which data were sought (e.g. participant and intervention characteristics, funding sources). Describe any assumptions made about any missing or unclear information. | Line 144-152 |
| **Study risk of bias assessment** | 11 | Specify the methods used to assess risk of bias in the included studies, including details of the tool(s) used, how many reviewers assessed each study and whether they worked independently, and if applicable, details of automation tools used in the process. | Line 144-146 |
| **Effect measures** | 12 | Specify for each outcome the effect measure(s) (e.g. risk ratio, mean difference) used in the synthesis or presentation of results. | Line 154-169 |
| **Synthesis methods** | 13a | Describe the processes used to decide which studies were eligible for each synthesis (e.g. tabulating the study intervention characteristics and comparing against the planned groups for each synthesis (item 5)). | Line 154-155 |
|  | 13b | Describe any methods required to prepare the data for presentation or synthesis, such as handling of missing summary statistics, or data conversions. | Line 154-178 |
|  | 13c | Describe any methods used to tabulate or visually display results of individual studies and syntheses. | Line 162-178 |
|  | 13d | Describe any methods used to synthesize results and provide a rationale for the choice(s). If meta-analysis was performed, describe the model(s), method(s) to identify the presence and extent of statistical heterogeneity, and software package(s) used. | Line 169-178 |
|  | 13e | Describe any methods used to explore possible causes of heterogeneity among study results (e.g. subgroup analysis, meta-regression). | Line 154-157 |
|  | 13f | Describe any sensitivity analyses conducted to assess robustness of the synthesized results. | Line 170-172 |
| **Reporting bias assessment** | 14 | Describe any methods used to assess risk of bias due to missing results in a synthesis (arising from reporting biases). | Line 170-172 |
| **Certainty assessment** | 15 | Describe any methods used to assess certainty (or confidence) in the body of evidence for an outcome. | Line 172-175 |
| **RESULTS** |  |  |  |
| **Study selection** | 16a | Describe the results of the search and selection process, from the number of records identified in the search to the number of studies included in the review, ideally using a flow diagram. | Line 182-189 |
|  | 16b | Cite studies that might appear to meet the inclusion criteria, but which were excluded, and explain why they were excluded. | Line 189-191 |
| **Study characteristics** | 17 | Cite each included study and present its characteristics. | Line 182-342, supplementary tables 1-9 |
| **Risk of bias in studies** | 18 | Present assessments of risk of bias for each included study. | Not shown |
| **Results of individual studies** | 19 | For all outcomes, present, for each study: (a) summary statistics for each group (where appropriate) and (b) an effect estimate and its precision (e.g. confidence/credible interval), ideally using structured tables or plots. | Line 208-213, Figure 2-7 |
| **Results of syntheses** | 20a | For each synthesis, briefly summarise the characteristics and risk of bias among contributing studies. | Figures 2-7 |
|  | 20b | Present results of all statistical syntheses conducted. If meta-analysis was done, present for each the summary estimate and its precision (e.g. confidence/credible interval) and measures of statistical heterogeneity. If comparing groups, describe the direction of the effect. | Line 354-400 |
|  | 20c | Present results of all investigations of possible causes of heterogeneity among study results. | Line 368-283 |
|  | 20d | Present results of all sensitivity analyses conducted to assess the robustness of the synthesized results. | Line 363-365 |
| **Reporting biases** | 21 | Present assessments of risk of bias due to missing results (arising from reporting biases) for each synthesis assessed. | Line 422-423 |
| **Certainty of evidence** | 22 | Present assessments of certainty (or confidence) in the body of evidence for each outcome assessed. | Line 358-385, Figures 6 and 7 |
| **DISCUSSION** |  |  |  |
| **Discussion** | 23a | Provide a general interpretation of the results in the context of other evidence. | Line 420-467 |
|  | 23b | Discuss any limitations of the evidence included in the review. | Line 469-473 |
|  | 23c | Discuss any limitations of the review processes used. | Line 473-481 |
|  | 23d | Discuss implications of the results for practice, policy, and future research. | Line 483-491 |
| **OTHER INFORMATION** |  |  |  |
| **Registration and protocol** | 24a | Provide registration information for the review, including register name and registration number, or state that the review was not registered. | Line 114-116 |
|  | 24b | Indicate where the review protocol can be accessed, or state that a protocol was not prepared. | Line 114-116 |
|  | 24c | Describe and explain any amendments to information provided at registration or in the protocol. | NA |
| **Support** | 25 | Describe sources of financial or non-financial support for the review, and the role of the funders or sponsors in the review. | Line 576-577 |
| **Competing interests** | 26 | Declare any competing interests of review authors. | Line 565-566 |
| **Availability of data, code and other materials** | 27 | Report which of the following are publicly available and where they can be found: template data collection forms; data extracted from included studies; data used for all analyses; analytic code; any other materials used in the review. | Supplementary material |

#####

# PRIMSA Abstract Checklist

| **Topic** | **No.** | **Item** | **Reported?** |
| --- | --- | --- | --- |
| **TITLE** |  |  |  |
| **Title** | 1 | Identify the report as a systematic review. | No |
| **BACKGROUND** |  |  |  |
| **Objectives** | 2 | Provide an explicit statement of the main objective(s) or question(s) the review addresses. | No |
| **METHODS** |  |  |  |
| **Eligibility criteria** | 3 | Specify the inclusion and exclusion criteria for the review. | Yes |
| **Information sources** | 4 | Specify the information sources (e.g. databases, registers) used to identify studies and the date when each was last searched. | No |
| **Risk of bias** | 5 | Specify the methods used to assess risk of bias in the included studies. | Yes |
| **Synthesis of results** | 6 | Specify the methods used to present and synthesize results. | Yes |
| **RESULTS** |  |  |  |
| **Included studies** | 7 | Give the total number of included studies and participants and summarise relevant characteristics of studies. | Yes |
| **Synthesis of results** | 8 | Present results for main outcomes, preferably indicating the number of included studies and participants for each. If meta-analysis was done, report the summary estimate and confidence/credible interval. If comparing groups, indicate the direction of the effect (i.e. which group is favoured). | Yes |
| **DISCUSSION** |  |  |  |
| **Limitations of evidence** | 9 | Provide a brief summary of the limitations of the evidence included in the review (e.g. study risk of bias, inconsistency and imprecision). | Yes |
| **Interpretation** | 10 | Provide a general interpretation of the results and important implications. | Yes |
| **OTHER** |  |  |  |
| **Funding** | 11 | Specify the primary source of funding for the review. | Yes |
| **Registration** | 12 | Provide the register name and registration number. | Yes |

*From:* Page MJ, McKenzie JE, Bossuyt PM, Boutron I, Hoffmann TC, Mulrow CD, et al. The PRISMA 2020 statement: an updated guideline for reporting systematic reviews. MetaArXiv. 2020, September 14. DOI: 10.31222/osf.io/v7gm2.

**Figure S1: Forest plot of the risks factor associated with ESBL Enterobacterales (random effects model-1)**

**
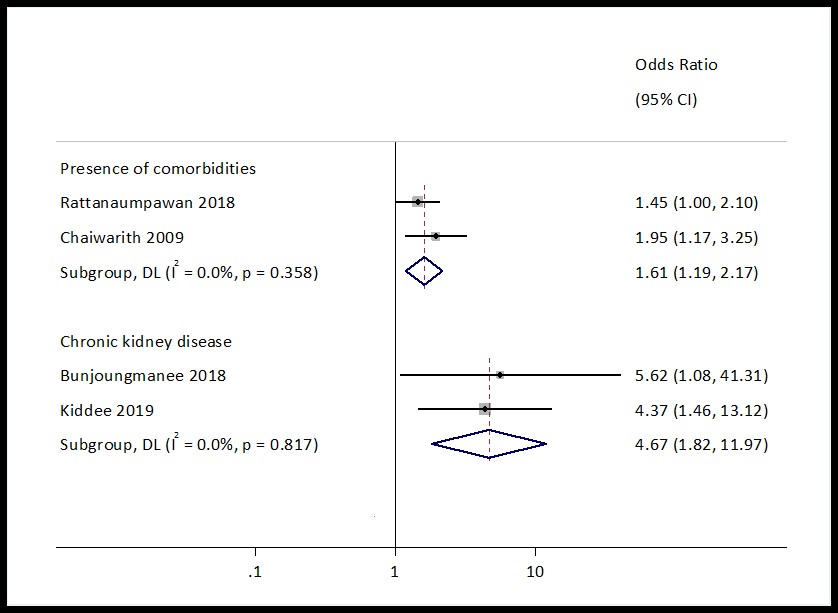
**

**Figure S2: Forest plot of the risks factor associated with ESBL Enterobacterales (random effects model-2)**

**
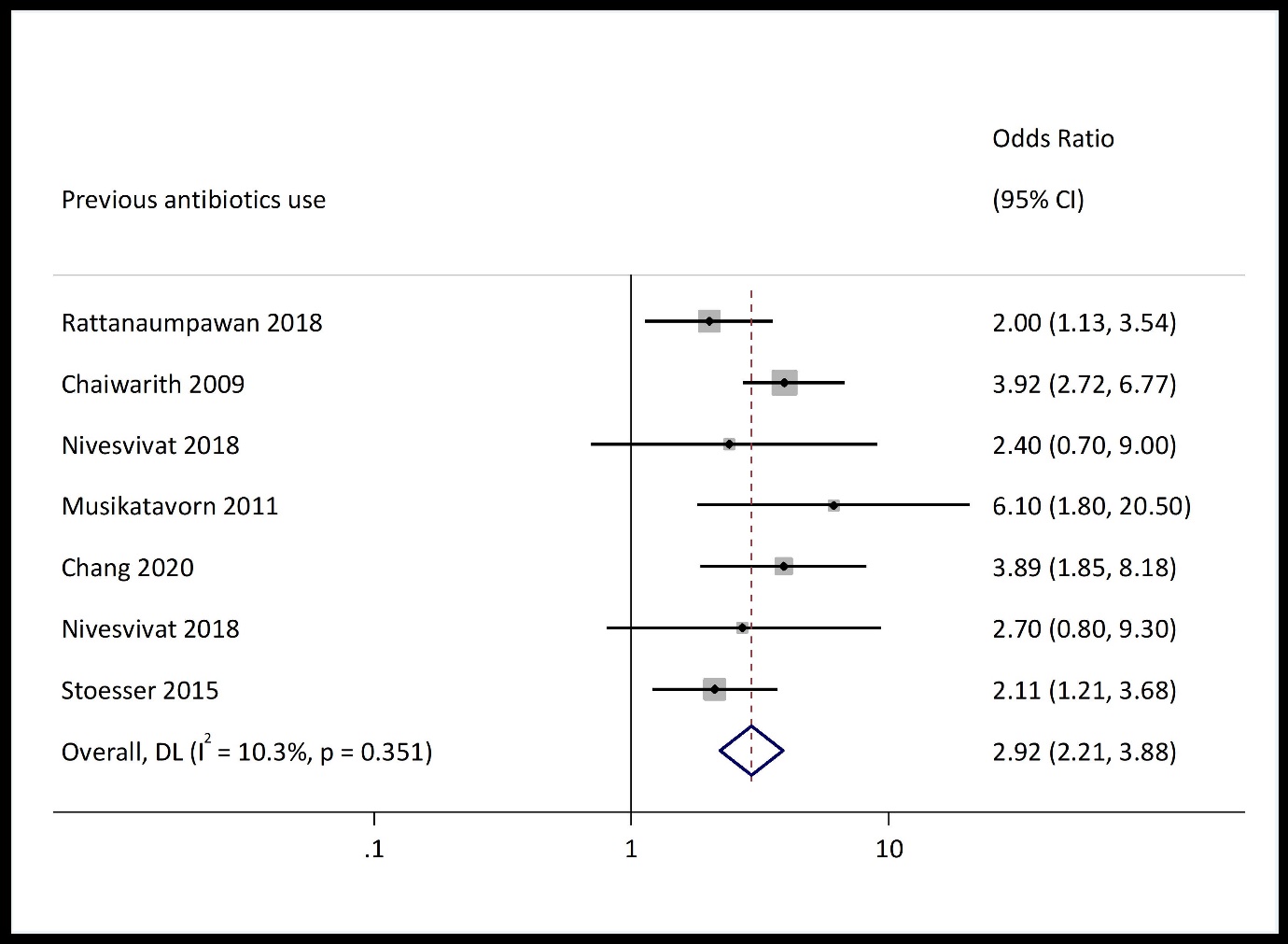
**

**Figure S3: Forest plot of the risks factor associated with ESBL *E. coli* (fixed effects model)**

**
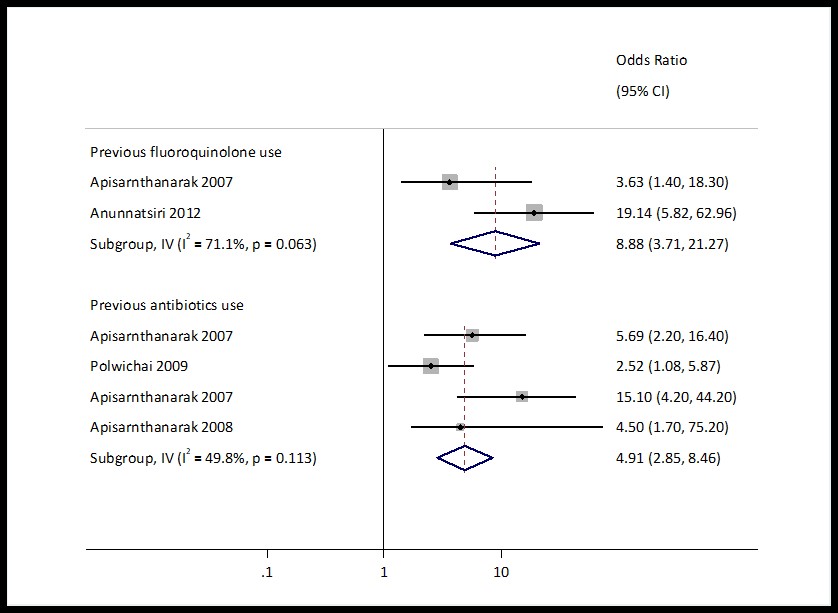
**

References:

Anunnatsiri, S., Towiwat, P., and Chaimanee, P. (2012). Risk factors and clinical outcomes of extended spectrum beta-lactamase (ESBL)-producing Escherichia coli septicemia at Srinagarind University Hospital, Thailand. *Southeast Asian Journal of Tropical Medicineand Public Health* 43(5)**,** 1169.

Apisarnthanarak, A., Kiratisin, P., and Mundy, L.M. (2008). Clinical and Molecular Epidemiology of Healthcare-Associated Infections Due to Extended-Spectrum β-Lactamase (ESBL)–Producing Strains of Escherichia coli and Klebsiella pneumoniae That Harbor Multiple ESBL Genes. *Infection Control & Hospital Epidemiology* 29(11)**,** 1026-1034.

Apisarnthanarak, A., Kiratisin, P., Saifon, P., Kitphati, R., Dejsirilert, S., and Mundy, L.M. (2007). Clinical and molecular epidemiology of community-onset, extended-spectrum β-lactamase-producing Escherichia coli infections in Thailand: A case-case-control study. *American journal of infection control* 35(9)**,** 606-612.

Atterby, C., Osbjer, K., Tepper, V., Rajala, E., Hernandez, J., Seng, S., et al. (2019). Carriage of carbapenemase‐ and extended‐spectrum cephalosporinase‐producing Escherichia coli and Klebsiella pneumoniae in humans and livestock in rural Cambodia; gender and age differences and detection of blaOXA‐48 in humans. *Zoonoses and public health* 66(6)**,** 603-617. doi: 10.1111/zph.12612.

Aung, M.S., San, N., Maw, W.W., San, T., Urushibara, N., Kawaguchiya, M., et al. (2018). Prevalence of extended-spectrum beta-lactamase and carbapenemase genes in clinical isolates of Escherichia coli in Myanmar: dominance of bla NDM-5 and emergence of bla OXA-181. *Microbial Drug Resistance* 24(9)**,** 1333-1344.

Aung, M.S., Win, N.C., San, N., Hlaing, M.S., Myint, Y.Y., Thu, P.P., et al. (2021). Prevalence of Extended-Spectrum Beta-Lactamase/Carbapenemase Genes and Quinolone-Resistance Determinants in Klebsiella pneumoniae Clinical Isolates from Respiratory Infections in Myanmar. *Microbial Drug Resistance* 27(1)**,** 36-43.

Barreto Miranda, I., Ignatius, R., Pfüller, R., Friedrich-Jänicke, B., Steiner, F., Paland, M., et al. (2016). High carriage rate of ESBL-producing Enterobacteriaceae at presentation and follow-up among travellers with gastrointestinal complaints returning from India and Southeast Asia. *Journal of travel medicine U6 - ctx_ver=Z39.88-2004&ctx_enc=info%3Aofi%2Fenc%3AUTF-8&rfr_id=info%3Asid%2Fsummon.serialssolutions.com&rft_val_fmt=info%3Aofi%2Ffmt%3Akev%3Amtx%3Ajournal&rft.genre=article&rft.atitle=High+carriage+rate+of+ESBL-producing+Enterobacteriaceae+at+presentation+and+follow-up+among+travellers+with+gastrointestinal+complaints+returning+from+India+and+Southeast+Asia&rft.jtitle=Journal+of+travel+medicine&rft.date=2016-02-01&rft.eissn=1708-8305&rft.volume=23&rft.issue=2&rft.spage=tav024&rft.epage=tav024&rft_id=info:doi/10.1093%2Fjtm%2Ftav024&rft.externalDBID=NO_FULL_TEXT&paramdict=en-US U7 - Journal Article* 23(2)**,** tav024-tav024. doi: 10.1093/jtm/tav024.

Berglund, B., Hoang, N.T.B., Tärnberg, M., Le, N.K., Welander, J., Nilsson, M., et al. (2018). Colistin- and carbapenem-resistant Klebsiella pneumoniae carrying mcr-1 and blaOXA-48 isolated at a paediatric hospital in Vietnam. *Journal of antimicrobial chemotherapy* 73(4)**,** 1100-1102. doi: 10.1093/jac/dkx491.

Biedenbach, D.J., Bouchillon, S.K., Hoban, D.J., Hackel, M., Phuong, D.M., Nga, T.T.T., et al. (2014). Antimicrobial susceptibility and extended-spectrum beta-lactamase rates in aerobic gram-negative bacteria causing intra-abdominal infections in Vietnam: report from the Study for Monitoring Antimicrobial Resistance Trends (SMART 2009–2011). *Diagnostic microbiology and infectious disease* 79(4)**,** 463-467. doi: 10.1016/j.diagmicrobio.2014.05.009.

Breurec, S., Guessennd, N., Timinouni, M., Le, T.T.H., Cao, V., Ngandjio, A., et al. (2013). Klebsiella pneumoniae resistant to third-generation cephalosporins in five African and two Vietnamese major towns: multiclonal population structure with two major international clonal groups, CG15 and CG258. *Clinical microbiology and infection* 19(4)**,** 349-355. doi: 10.1111/j.1469-0691.2012.03805.x.

Bubpamala, J., Khuntayaporn, P., Thirapanmethee, K., Montakantikul, P., Santanirand, P., and Chomnawang, M.T. (2018). Phenotypic and genotypic characterizations of extended-spectrum beta-lactamase-producing Escherichia coli in Thailand. *Infection and drug resistance* 11**,** 2151-2157. doi: 10.2147/IDR.S174506.

Bui, T.K.N., Bui, T.M.H., Ueda, S., Le, D.T., Yamamoto, Y., and Hirai, I. (2018). Potential transmission opportunity of CTX-M-producing Escherichia coli on a large-scale chicken farm in Vietnam. *Journal of global antimicrobial resistance* 13**,** 1-6.

Cao, V., Lambert, T., Nhu, D.Q., Loan, H.K., Hoang, N.K., Arlet, G., et al. (2002). Distribution of Extended-Spectrum β-Lactamases in Clinical Isolates of Enterobacteriaceae in Vietnam. *Antimicrobial Agents and Chemotherapy* 46(12)**,** 3739-3743. doi: 10.1128/AAC.46.12.3739-3743.2002.

Caron, Y., Chheang, R., Puthea, N., Soda, M., Boyer, S., Tarantola, A., et al. (2018). Beta-lactam resistance among Enterobacteriaceae in Cambodia: The four-year itch. *International journal of infectious diseases* 66(C)**,** 74-79. doi: 10.1016/j.ijid.2017.10.025.

Cha, M.K., Kang, C.-I., Kim, S.H., Thamlikitkul, V., So, T.M.-k., Ha, Y.E., et al. (2017). Emergence and Dissemination of ST131 Escherichia coli Isolates Among Patients with Hospital-Acquired Pneumonia in Asian Countries. *Microbial drug resistance (Larchmont, N.Y.)* 23(1)**,** 79-82. doi: 10.1089/mdr.2016.0009.

Chaiwarith, R., Pasogpakdee, P., Salee, P., Kanjanaratanakorn, K., Sirisanthana, T., and Supparatpinyo, K. (2008). Risk factors for extended-spectrum β-lactamase-producing Klebsiella pneumoniae and Escherichia coli acquisition in a tertiary care teaching hospital in Thailand. *The Journal of hospital infection* 71(3)**,** 285-286. doi: 10.1016/j.jhin.2008.11.021.

Chalmers, L., Cross, J., Chu, C.S., Phyo, A.P., Trip, M., Ling, C., et al. (2015). The role of point‐of‐care tests in antibiotic stewardship for urinary tract infections in a resource‐limited setting on the Thailand–Myanmar border. *Tropical medicine & international health* 20(10)**,** 1281-1289. doi: 10.1111/tmi.12541.

Chanawong, A., Lulitanond, A., Kaewkes, W., Lulitanond, V., Srigulbutr, S., and Homchampa, P. (2007). CTX-M extended-spectrum β-lactamases among clinical isolates of enterobacteriaceae in a thai university hospital. *Southeast Asian journal of tropical medicine and public health* 38(3)**,** 493-500.

Chanawong, A., M'Zali, F.H., Heritage, J., Lulitanond, A., and Hawkey, P.M. (2001). SHV-12, SHV-5, SHV-2a and VEB-1 extended-spectrum β-lactamases in Gram-negative bacteria isolated in a university hospital in Thailand. *Journal of antimicrobial chemotherapy* 48(6)**,** 839-852. doi: 10.1093/jac/48.6.839.

Chang, K., Rattanavong, S., Mayxay, M., Keoluangkhot, V., Davong, V., Vongsouvath, M., et al. (2020). Bacteremia Caused by Extended-Spectrum Beta-Lactamase–Producing Enterobacteriaceae in Vientiane, Lao PDR: A 5-Year Study. *The American journal of tropical medicine and hygiene* 102(5)**,** 1137-1143.

Chang, Y.-T., Coombs, G., Ling, T., Balaji, V., Rodrigues, C., Mikamo, H., et al. (2017). Epidemiology and trends in the antibiotic susceptibilities of Gram-negative bacilli isolated from patients with intra-abdominal infections in the Asia-Pacific region, 2010–2013. *International journal of antimicrobial agents* 49(6)**,** 734-739. doi: 10.1016/j.ijantimicag.2017.01.030.

Cusack, T.-P., Phimolsarnnousith, V., Duangmala, K., Phoumin, P., Turton, J., Hopkins, K.L., et al. (2019). Molecular characterization of carbapenem-resistant Escherichia coli and Acinetobacter baumannii in the Lao People’s Democratic Republic. *Journal of antimicrobial chemotherapy* 74(9)**,** 2810-2821. doi: 10.1093/jac/dkz234.

Dat, V.Q., Vu, H.N., The, H.d., Nguyen, H.T., Hoang, L.B., Viet, D.V.T., et al. (2017). Bacterial bloodstream infections in a tertiary infectious diseases hospital in Northern Vietnam: aetiology, drug resistance, and treatment outcome. *BMC infectious diseases* 17(1)**,** 493-493. doi: 10.1186/s12879-017-2582-7.

Girlich, D., Poirel, L., Leelaporn, A., Karim, A., Tribuddharat, C., Fennewald, M., et al. (2001). Molecular Epidemiology of the Integron-Located VEB-1 Extended-Spectrum β-Lactamase in Nosocomial Enterobacterial Isolates in Bangkok, Thailand. *Journal of Clinical Microbiology* 39(1)**,** 175-182. doi: 10.1128/JCM.39.1.175-182.2001.

Hawser, S.P., Bouchillon, S.K., Hoban, D.J., Badal, R.E., Hsueh, P.-R., and Paterson, D.L. (2009). Emergence of High Levels of Extended-Spectrum-β-Lactamase-Producing Gram-Negative Bacilli in the Asia-Pacific Region: Data from the Study for Monitoring Antimicrobial Resistance Trends (SMART) Program, 2007. *Antimicrobial Agents and Chemotherapy* 53(8)**,** 3280-3284. doi: 10.1128/AAC.00426-09.

Hoang, C.Q., Nguyen, H.D., Vu, H.Q., Nguyen, A.T., Pham, B.T., Tran, T.L., et al. (2019). Emergence of New Delhi Metallo-Beta-Lactamase (NDM) and Klebsiella pneumoniae Carbapenemase (KPC) Production by Escherichia coli and Klebsiella pneumoniae in Southern Vietnam and Appropriate Methods of Detection: A Cross-Sectional Study. *BioMed research international* 2019**,** 9757625-9757629. doi: 10.1155/2019/9757625.

Hoang, P.H., Awasthi, S.P., Nguyen, P.D.O., Nguyen, N.L.H., Nguyen, D.T.A., Le, N.H., et al. (2017a). Antimicrobial resistance profiles and molecular characterization of Escherichia coli strains isolated from healthy adults in Ho Chi Minh City, Vietnam. *Journal of veterinary medical science* 79(3)**,** 479-485. doi: 10.1292/jvms.16-0639.

Hoang, T.A.V., Hoang, T.A.V., Nguyen, T.N.H., Nguyen, T.N.H., Ueda, S., Ueda, S., et al. (2017b). Common findings of bla CTX-M-55-encoding 104–139 kbp plasmids harbored by extended-spectrum β-lactamase-producing Escherichia coli in pork meat, wholesale market workers, and patients with urinary tract infection in Vietnam. *Current microbiology* 74(2)**,** 203-211. doi: 10.1007/s00284-016-1174-x.

Hoang, T.H., Wertheim, H., Minh, N.B., Duong, T.N., Anh, D.D., Phuong, T.T.L., et al. (2013). Carbapenem-resistant Escherichia coli and Klebsiella pneumoniae strains containing New Delhi metallo-beta-lactamase isolated from two patients in Vietnam. *Journal of clinical microbiology* 51(1)**,** 373-374. doi: 10.1128/JCM.02322-12.

Honda, N.H., Aoki, K., Kamisasanuki, T., Matsuda, N., To, M., Matsushima, H., et al. (2019). Isolation of three distinct carbapenemase-producing Gram-negative bacteria from a Vietnamese medical tourist. *Journal of Infection and Chemotherapy* 25(10)**,** 811-815.

Hout, B., Oum, C., Men, P., Vanny, V., Supaprom, C., Heang, V., et al. (2015). Drug resistance in bacteria isolated from patients presenting with wounds at a non-profit Surgical Center in Phnom Penh, Cambodia from 2011-2013. *Tropical diseases, travel medicine and vaccines* 1(1)**,** 4-4. doi: 10.1186/s40794-015-0006-5.

Huang, C.-C., Chen, Y.-S., Toh, H.-S., Lee, Y.-L., Liu, Y.-M., Ho, C.-M., et al. (2012). Impact of revised CLSI breakpoints for susceptibility to third-generation cephalosporins and carbapenems among Enterobacteriaceae isolates in the Asia-Pacific region: results from the Study for Monitoring Antimicrobial Resistance Trends (SMART), 2002–2010. *International journal of antimicrobial agents* 40**,** S4-S10. doi: 10.1016/S0924-8579(12)70003-1.

Hung, P.N., Quyet, D., Thanh, K.C., Pho, D.C., Tien, T.V., Dung, Q.A., et al. (2019). Antibiotic Resistance Profile and Diversity of Subtypes Genes in Escherichia coli Causing Bloodstream Infection in Northern Vietnam. *Open Access Maced J Med Sci* 7(24)**,** 4393-4398. doi: 10.3889/oamjms.2019.842.

Jakobsen, L., Hammerum, A.M., Hansen, F., and Fuglsang-Damgaard, D. (2014). An ST405 NDM-4-producing Escherichia coli isolated from a Danish patient previously hospitalized in Vietnam. *Journal of antimicrobial chemotherapy* 69(2)**,** 559-560. doi: 10.1093/jac/dkt356.

Jean, S.-S., Coombs, G., Ling, T., Balaji, V., Rodrigues, C., Mikamo, H., et al. (2016). Epidemiology and antimicrobial susceptibility profiles of pathogens causing urinary tract infections in the Asia-Pacific region: Results from the Study for Monitoring Antimicrobial Resistance Trends (SMART), 2010–2013. *International journal of antimicrobial agents* 47(4)**,** 328-334. doi: 10.1016/j.ijantimicag.2016.01.008.

Jean, S.-S., Hsueh, P.-R., and Group, S.A.-P. (2017). Distribution of ESBLs, AmpC β-lactamases and carbapenemases among Enterobacteriaceae isolates causing intra-abdominal and urinary tract infections in the Asia-Pacific region during 2008-14: results from the Study for Monitoring Antimicrobial Resistance Trends (SMART). *Journal of antimicrobial chemotherapy* 72(1)**,** 166-171. doi: 10.1093/jac/dkw398.

Jean, S.-S., Lee, W.-S., Hsueh, P.-R., and Group, S.A.-P. (2018). Ertapenem non-susceptibility and independent predictors of the carbapenemase production among the Enterobacteriaceae isolates causing intra-abdominal infections in the Asia-Pacific region: results from the Study for Monitoring Antimicrobial Resistance Trends (SMART). *Infection and drug resistance* 11**,** 1881-1891. doi: 10.2147/IDR.S181085.

Jitsurong, S., and Yodsawat, J. (2006). Prevalence of extended-spectrum beta- lactamases (ESBLs) produced in blood isolates of gram-negative bacteria in a teaching hospital in southern Thailand. *Southeast Asian journal of tropical medicine and public health* 37(1)**,** 131-135.

Jones, S.L., Nguyen, V.K., Nguyen, T.M.P., and Athan, E. (2006). Prevalence of multiresistant Gram‐negative organisms in a surgical hospital in Ho Chi Minh Cit, Vietnam. *Tropical medicine & international health* 11(11)**,** 1725-1730. doi: 10.1111/j.1365-3156.2006.01731.x.

Kanoksil, M., Jatapai, A., Peacock, S.J., and Limmathurotsakul, D. (2013). Epidemiology, Microbiology and Mortality Associated with Community-Acquired Bacteremia in Northeast Thailand: A Multicenter Surveillance Study. *PloS one* 8(1)**,** e54714-e54714. doi: 10.1371/journal.pone.0054714.

Kazmierczak, K.M., Rabine, S., Hackel, M., McLaughlin, R.E., Biedenbach, D.J., Bouchillon, S.K., et al. (2016). Multiyear, Multinational Survey of the Incidence and Global Distribution of Metallo-β-Lactamase-Producing Enterobacteriaceae and Pseudomonas aeruginosa. *Antimicrobial agents and chemotherapy* 60(2)**,** 1067-1078. doi: 10.1128/AAC.02379-15.

Kerdsin, A., Deekae, S., Chayangsu, S., Hatrongjit, R., Chopjitt, P., Takeuchi, D., et al. (2019). Genomic characterization of an emerging bla KPC-2 carrying Enterobacteriaceae clinical isolates in Thailand. *Scientific reports* 9(1)**,** 1-7.

Kiddee, A., Assawatheptawee, K., Na-Udom, A., Boonsawang, P., Treebupachatsakul, P., Walsh, T.R., et al. (2019). Risk factors for extended-spectrum β-lactamase-producing enterobacteriaceae carriage in patients admitted to intensive care unit in a tertiary care hospital in Thailand. *Microbial Drug Resistance* 25(8)**,** 1182-1190.

Kiddee, A., Assawatheptawee, K., Na-Udom, A., Treebupachatsakul, P., Wangteeraprasert, A., Walsh, T.R., et al. (2018). Risk Factors for Gastrointestinal Colonization and Acquisition of Carbapenem-Resistant Gram-Negative Bacteria among Patients in Intensive Care Units in Thailand. *Antimicrobial agents and chemotherapy* 62(8). doi: 10.1128/AAC.00341-18.

Kiratisin, P., Apisarnthanarak, A., Laesripa, C., and Saifon, P. (2008a). Molecular characterization and epidemiology of extended-spectrum-β-lactamase-producing Escherichia coli and Klebsiella pneumoniae isolates causing health care-associated infection in Thailand, where the CTX-M family is endemic. *Antimicrobial agents and chemotherapy* 52(8)**,** 2818-2824.

Kiratisin, P., Apisarnthanarak, A., Saifon, P., Laesripa, C., Kitphati, R., and Mundy, L.M. (2007). The emergence of a novel ceftazidime-resistant CTX-M extended-spectrum β-lactamase, CTX-M-55, in both community-onset and hospital-acquired infections in Thailand. *Diagnostic microbiology and infectious disease* 58(3)**,** 349-355. doi: 10.1016/j.diagmicrobio.2007.02.005.

Kiratisin, P., Chattammanat, S., Sa-Nguansai, S., Dansubutra, B., Nangpatharapornthawee, P., Patthamalai, P., et al. (2008b). A 2-year trend of extended-spectrum β-lactamase-producing Escherichia coli and Klebsiella pneumoniae in Thailand: an alert for infection control. *Transactions of the Royal Society of Tropical Medicine and Hygiene* 102(5)**,** 460-464. doi: 10.1016/j.trstmh.2008.02.005.

Kiratisin, P., Chongthaleong, A., Tan, T.Y., Lagamayo, E., Roberts, S., Garcia, J., et al. (2012). Comparative in vitro activity of carbapenems against major Gram-negative pathogens: results of Asia-Pacific surveillance from the COMPACT II study. *International journal of antimicrobial agents* 39(4)**,** 311-316. doi: 10.1016/j.ijantimicag.2012.01.002.

Kusum, M., Wongwanich, S., Dhiraputra, C., Pongpech, P., and Naenna, P. (2004). Occurrence of extended-spectrum beta-lactamase in clinical isolates of Klebsiella pneumoniae in a University Hospital, Thailand. *JOURNAL-MEDICAL ASSOCIATION OF THAILAND* 87(9)**,** 1029-1033.

L, L., Lk, T., Td, L.-H., Bp, T., Hn, L.-V., Yn, N., et al. (2019). Coexistence Of Plasmid-Mediated mcr-1 And blaNDM-4 Genes In A Klebsiella pneumoniae Clinical Strain In Vietnam. *Infection and drug resistance* 12**,** 3703-3707.

Lan, N.P.H., Hien, N.H., Le Thi Phuong, T., Thanh, D.P., Thieu, N.T.V., Ngoc, D.T.T., et al. (2017). Phenotypic and genotypic characteristics of ESBL and AmpC producing organisms associated with bacteraemia in Ho Chi Minh City, Vietnam. *Antimicrobial resistance & infection control* 6(1)**,** 105-105. doi: 10.1186/s13756-017-0265-1.

Laolerd, W., Akeda, Y., Preeyanon, L., Ratthawongjirakul, P., and Santanirand, P. (2018). Carbapenemase-Producing Carbapenem-Resistant Enterobacteriaceae from Bangkok, Thailand, and Their Detection by the Carba NP and Modified Carbapenem Inactivation Method Tests. *Microbial drug resistance (Larchmont, N.Y.)* 24(7)**,** 16-1011. doi: 10.1089/mdr.2018.0080.

Lunha, K., Chanawong, A., Lulitanond, A., Wilailuckana, C., Charoensri, N., Wonglakorn, L., et al. (2015). High-level carbapenem-resistant OXA-48-producing Klebsiella pneumoniae with a novel OmpK36 variant and low-level carbapenem-resistant non-porin-deficient OXA-181-producing Escherichia coli from Thailand. *Diagnostic microbiology and infectious disease* 85(2)**,** 221-226. doi: 10.1016/j.diagmicrobio.2016.03.009.

Luvsansharav, U.-O., Hirai, I., Yamamoto, Y., Niki, M., Sasaki, T., Makimoto, K., et al. (2011). Analysis of risk factors for a high prevalence of extended-spectrum β-lactamase-producing Enterobacteriaceae in asymptomatic individuals in rural Thailand. *Journal of medical microbiology* 60(5)**,** 619-624. doi: 10.1099/jmm.0.026955-0.

Matsumura, Y., Peirano, G., Motyl, M.R., Adams, M.D., Chen, L., Kreiswirth, B., et al. (2017). Global Molecular Epidemiology of IMP-Producing Enterobacteriaceae. *Antimicrobial agents and chemotherapy* 61(4). doi: 10.1128/AAC.02729-16.

Moore, C.E., Sona, S., Poda, S., Putchhat, H., Kumar, V., Sopheary, S., et al. (2016). Antimicrobial susceptibility of uropathogens isolated from Cambodian children. *Paediatrics and international child health* 36(2)**,** 113-117. doi: 10.1179/2046905515Y.0000000008.

Musikatavorn, K., Chumpengpan, C., and Sujinpram, C. (2011). Risk factors of extended-spectrum beta-lactamaseproducing Enterobacteriaceae bacteremia in Thai emergency department: a retrospective case-control study. *Asian Biomedicine* 5(1)**,** 129-138.

Myat, T.O., Hannaway, R.F., Zin, K.N., Htike, W.W., Win, K.K., Crump, J.A., et al. (2017). ESBL- and Carbapenemase-Producing Enterobacteriaceae in Patients with Bacteremia, Yangon, Myanmar, 2014. *Emerging infectious diseases* 23(5)**,** 857-859. doi: 10.3201/eid2305.161100.

Myat, T.O., Oo, K.M., Mone, H.K., Htike, W.W., Biswas, A., Hannaway, R.F., et al. (2020). A prospective study of bloodstream infections among febrile adolescents and adults attending Yangon General Hospital, Yangon, Myanmar. *PLoS neglected tropical diseases* 14(4)**,** e0008268-e0008268. doi: 10.1371/journal.pntd.0008268.

Nadimpalli, M.L., de Lauzanne, A., Phe, T., Borand, L., Jacobs, J., Fabre, L., et al. (2019). Escherichia coli ST410 among humans and the environment in Southeast Asia. *International journal of antimicrobial agents* 54(2)**,** 228-232. doi: 10.1016/j.ijantimicag.2019.05.024.

Netikul, T., and Kiratisin, P. (2015). Genetic Characterization of Carbapenem-Resistant Enterobacteriaceae and the Spread of Carbapenem-Resistant Klebsiella pneumonia ST340 at a University Hospital in Thailand. *PloS one* 10(9)**,** e0139116-e0139116. doi: 10.1371/journal.pone.0139116.

Netikul, T., Sidjabat, H.E., Paterson, D.L., Kamolvit, W., Tantisiriwat, W., Steen, J.A., et al. (2014). Characterization of an IncN2-type bla NDM-1-carrying plasmid in Escherichia coli ST131 and Klebsiella pneumoniae ST11 and ST15 isolates in Thailand. *Journal of Antimicrobial Chemotherapy* 69(11)**,** 3161-3163.

Nirdnoy, W., Mason, C.J., Kietsiri, P., Sethabutr, O., Poramathikul, K., Ruksasiri, S., et al. (2017). PREVALENCE OF β-LACTAMASES IN ENTEROTOXIGENIC ESCHERICHIA COLI CLINICAL ISOLATES COLLECTED IN CAMBODIA, KENYA, NEPAL, THAILAND, UZBEKISTAN, AND VIETNAM, FROM 2001 TO 2010. *Southeast Asian journal of tropical medicine and public health* 48(3)**,** 590.

Niumsup, P.R., Niumsup, P.R., Tansawai, U., Tansawai, U., Na-udom, A., Na-udom, A., et al. (2018). Prevalence and risk factors for intestinal carriage of CTX-M-type ESBLs in Enterobacteriaceae from a Thai community. *European journal of clinical microbiology & infectious diseases* 37(1)**,** 69-75. doi: 10.1007/s10096-017-3102-9.

Niumsup, P.R., Tansawai, U., Boonkerd, N., Polwichai, P., and Dejsirilert, S. (2008). Dissemination of extended-spectrum β-lactamase-producing Klebsiella pneumoniae and Escherichia coli in Thai hospitals. *Journal of infection and chemotherapy : official journal of the Japan Society of Chemotherapy* 14(6)**,** 404-408. doi: 10.1007/s10156-008-0642-Z.

Nivesvivat, T., Piyaraj, P., Thunyaharn, S., Watanaveeradej, V., and Suwanpakdee, D. (2018). Clinical epidemiology, risk factors and treatment outcomes of extended-spectrum beta-lactamase producing Enterobacteriaceae bacteremia among children in a Tertiary Care Hospital, Bangkok, Thailand. *BMC research notes* 11(1)**,** 624-624. doi: 10.1186/s13104-018-3729-3.

Paveenkittiporn, W., Kerdsin, A., Chokngam, S., Bunthi, C., Sangkitporn, S., and Gregory, C.J. (2016). Emergence of plasmid-mediated colistin resistance and New Delhi metallo-β-lactamase genes in extensively drug-resistant Escherichia coli isolated from a patient in Thailand. *Diagnostic microbiology and infectious disease* 87(2)**,** 157-159. doi: 10.1016/j.diagmicrobio.2016.11.005.

Phongpaichit, S., Tunyapanit, W., and Pruekprasert, P. (2011). Antimicrobial Resistance, Class 1 Integrons and Extended-Spectrum β-Lactamases in Escherichia coli Clinical Isolates from Patients in South Thailand. *Journal of health science (Tokyo, Japan)* 57(3)**,** 281-288. doi: 10.1248/jhs.57.281.

Polwichai, P., Trakulsomboon, S., Dejsirilert, S., Thongmali, O., Sawanpanyalert, P., Aswapokee, N., et al. (2011). Long-term study of Escherichia coli and Klebsiella pneumoniae isolates producing extended-spectrum beta-lactamases. *Journal of the Medical Association of Thailand* 92(8)**,** 53.

Pongpech, P., Naenna, P., Taipobsakul, Y., Tribuddharat, C., and Srifuengfung, S. (2008). Prevalence of extended-spectrum beta-lactamase and class 1 integron integrase gene intl1 in Escherichia coli from Thai patients and healthy adults. *Southeast Asian journal of tropical medicine and public health* 39(3)**,** 425-433.

Pornsinchai, P., Chongtrakool, P., Diraphat, P., Siripanichgon, K., and Malathum, K. (2015). EMERGENCY ROOM: AN UNRECOGNIZED SOURCE OF EXTENDED-SPECTRUM [Beta]-LACTAMASE PRODUCING ESCHERICHIA COLI AND KLEBSIELLA PNEUMONIAE. *Southeast Asian journal of tropical medicine and public health* 46(1)**,** 51.

Preechachuawong, P., Santimaleeworagun, W., Jitwasinkul, T., and Samret, W. (2015). DETECTION OF NEW DELHI METALLO-BETA-LACTAMASE-1-PRODUCING KLEBSIELLA PNEUMONIAE AT A GENERAL HOSPITAL IN THAILAND. *Southeast Asian journal of tropical medicine and public health* 46(6)**,** 1031-1036.

Rammaert, B., Goyet, S., Beauté, J., Hem, S., Te, V., Try, P.L., et al. (2012). Klebsiella pneumoniae related community-acquired acute lower respiratory infections in Cambodia: clinical characteristics and treatment. *BMC infectious diseases* 12(1)**,** 3-3. doi: 10.1186/1471-2334-12-3.

Reechaipichitkul, W., Phondongnok, S., Bourpoern, J., and Chaimanee, P. (2013). Causative agents and resistance among hospital-acquired and ventilator-associated pneumonia patients at Srinagarind Hospital, northeastern Thailand. *Southeast Asian journal of tropical medicine and public health* 44(3)**,** 490-502.

Rhodes, J., Jorakate, P., Makprasert, S., Sangwichian, O., Kaewpan, A., Akarachotpong, T., et al. (2019). Population-based bloodstream infection surveillance in rural Thailand, 2007-2014. *BMC public health* 19(Suppl 3)**,** 521-521. doi: 10.1186/s12889-019-6775-4.

Rimrang, B., Chanawong, A., Lulitanond, A., Wilailuckana, C., Charoensri, N., Sribenjalux, P., et al. (2012). Emergence of NDM-1- and IMP-14a-producing Enterobacteriaceae in Thailand. *Journal of antimicrobial chemotherapy* 67(11)**,** 2626-2630. doi: 10.1093/jac/dks267.

Runcharoen, C., Moradigaravand, D., Blane, B., Paksanont, S., Thammachote, J., Anun, S., et al. (2017a). Whole genome sequencing reveals high-resolution epidemiological links between clinical and environmental Klebsiella pneumoniae. *Genome medicine* 9(1)**,** 6-6. doi: 10.1186/s13073-017-0397-1.

Runcharoen, C., Raven, K.E., Reuter, S., Kallonen, T., Paksanont, S., Thammachote, J., et al. (2017b). Whole genome sequencing of ESBL-producing Escherichia coli isolated from patients, farm waste and canals in Thailand. *Genome medicine* 9(1)**,** 81-81. doi: 10.1186/s13073-017-0471-8.

Ruppé, E., Hem, S., Lath, S., Gautier, V., Ariey, F., Sarthou, J.-L., et al. (2009). CTX-M β-lactamases in Escherichia coli from community-acquired urinary tract infections, Cambodia. *Emerging infectious diseases* 15(5)**,** 741.

San, T., Moe, I., Ashley, E.A., and San, N. (2021). High burden of infections caused by ESBL-producing MDR Escherichia coli in paediatric patients, Yangon, Myanmar. *JAC Antimicrob Resist* 3(1)**,** dlab011. doi: 10.1093/jacamr/dlab011.

Sasaki, T., Hira, I., Niki, M., Nakamura, T., Komalamisra, C., Maipanich, W., et al. (2010). High prevalence of CTX-M β-lactamase-producing Enterobacteriaceae in stool specimens obtained from healthy individuals in Thailand. *Journal of antimicrobial chemotherapy* 65(4)**,** 666-668. doi: 10.1093/jac/dkq008.

Sawatwong, P., Sapchookul, P., Whistler, T., Gregory, C.J., Sangwichian, O., Makprasert, S., et al. (2019). High Burden of Extended-Spectrum β-Lactamase-Producing Escherichia coli and Klebsiella pneumoniae Bacteremia in Older Adults: A Seven-Year Study in Two Rural Thai Provinces. *The American journal of tropical medicine and hygiene* 100(4)**,** 943-951. doi: 10.4269/ajtmh.18-0394.

Seenama, C., Thamlikitkul, V., and Ratthawongjirakul, P. (2019). Multilocus sequence typing and bla ESBL characterization of extended-spectrum beta-lactamase-producing Escherichia coli isolated from healthy humans and swine in Northern Thailand. *Infection and drug resistance* 12**,** 2201-2214. doi: 10.2147/IDR.S209545.

Sethaphanich, N., Santanirand, P., Rattanasiri, S., Techasaensiri, C., Chaisavaneeyakorn, S., and Apiwattanakul, N. (2016). Pediatric extended spectrum β‐lactamase infection: Community‐acquired infection and treatment options. *Pediatrics international* 58(5)**,** 338-346. doi: 10.1111/ped.12845.

Sheng, W.-H., Badal, R.E., Hsueh, P.-R., and Program, S. (2013). Distribution of Extended-Spectrum β-Lactamases, AmpC β-Lactamases, and Carbapenemases among Enterobacteriaceae Isolates Causing Intra-Abdominal Infections in the Asia-Pacific Region: Results of the Study for Monitoring Antimicrobial Resistance Trends (SMART). *Antimicrobial Agents and Chemotherapy* 57(7)**,** 2981-2988. doi: 10.1128/AAC.00971-12.

Srijan, A., Margulieux, K.R., Ruekit, S., Snesrud, E., Maybank, R., Serichantalergs, O., et al. (2018). Genomic Characterization of Nonclonal mcr-1-Positive Multidrug-Resistant Klebsiella pneumoniae from Clinical Samples in Thailand. *Microbial drug resistance (Larchmont, N.Y.)* 24(4)**,** 43-410. doi: 10.1089/mdr.2017.0400.

Stoesser, N., Sheppard, A.E., Moore, C.E., Golubchik, T., Parry, C.M., Nget, P., et al. (2015a). Extensive Within-Host Diversity in Fecally Carried Extended-Spectrum-Beta-Lactamase-Producing Escherichia coli Isolates: Implications for Transmission Analyses. *Journal of clinical microbiology* 53(7)**,** 2122-2131. doi: 10.1128/JCM.00378-15.

Stoesser, N., Sheppard, A.E., Peirano, G., Sebra, R.P., Lynch, T., Anson, L.W., et al. (2016). First Report of blaIMP-14 on a Plasmid Harboring Multiple Drug Resistance Genes in Escherichia coli Sequence Type 131. *Antimicrobial agents and chemotherapy U6 - ctx_ver=Z39.88-2004&ctx_enc=info%3Aofi%2Fenc%3AUTF-8&rfr_id=info%3Asid%2Fsummon.serialssolutions.com&rft_val_fmt=info%3Aofi%2Ffmt%3Akev%3Amtx%3Ajournal&rft.genre=article&rft.atitle=First+Report+of+blaIMP-14+on+a+Plasmid+Harboring+Multiple+Drug+Resistance+Genes+in+Escherichia+coli+Sequence+Type+131&rft.jtitle=Antimicrobial+agents+and+chemotherapy&rft.date=2016-08-01&rft.eissn=1098-6596&rft.volume=60&rft.issue=8&rft.spage=5068&rft.epage=5071&rft_id=info:doi/10.1128%2FAAC.00840-16&rft.externalDBID=NO_FULL_TEXT&paramdict=en-US U7 - Journal Article* 60(8)**,** 5068-5071. doi: 10.1128/AAC.00840-16.

Stoesser, N., Xayaheuang, S., Vongsouvath, M., Phommasone, K., Elliott, I., Del Ojo Elias, C., et al. (2015b). Colonization with Enterobacteriaceae producing ESBLs in children attending pre-school childcare facilities in the Lao People's Democratic Republic. *Journal of antimicrobial chemotherapy* 70(6)**,** 1893-1897. doi: 10.1093/jac/dkv021.

Sugawara, Y., Akeda, Y., Sakamoto, N., Takeuchi, D., Motooka, D., Nakamura, S., et al. (2017). Genetic characterization of blaNDM-harboring plasmids in carbapenem-resistant Escherichia coli from Myanmar. *PloS one* 12(9)**,** e0184720-e0184720. doi: 10.1371/journal.pone.0184720.

Sukkua, K., Rattanachuay, P., Khianngam, S., Hayeebilan, F., and Sukhumungoon, P. (2019). MOLECULAR CHARACTERIZATION OF EXTRAINTESTINAL PATHOGENIC ESCHERICHIA COLI FROM HUMANS IN SOUTHERN THAILAND. *SOUTHEAST ASIAN JOURNAL OF TROPICAL MEDICINE AND PUBLIC HEALTH* 50(5)**,** 860-870.

Tada, T., Tsuchiya, M., Shimada, K., Nga, T.T.T., Thu, L.T.A., Phu, T.T., et al. (2017). Dissemination of Carbapenem-resistant Klebsiella pneumoniae clinical isolates with various combinations of Carbapenemases (KPC-2, NDM-1, NDM-4, and OXA-48) and 16S rRNA Methylases (RmtB and RmtC) in Vietnam. *BMC infectious diseases* 17(1)**,** 467-467. doi: 10.1186/s12879-017-2570-y.

Tantracheewathorn, T., Vititpatarapak, N., and Phumisantiphong, U. (2007). Epidemiologic study of nosocomial bacterial infection of pediatric patients at BMA Medical College and Vajira Hospital. *JOURNAL-MEDICAL ASSOCIATION OF THAILAND* 90(2)**,** 258.

Tham, J., Odenholt, I., Walder, M., Brolund, A., Ahl, J., and Melander, E. (2010). Extended-spectrum beta-lactamase-producing Escherichia coli in patients with travellers' diarrhoea. *Scandinavian journal of infectious diseases* 42(4)**,** 275-280. doi: 10.3109/00365540903493715.

Themphachana, M., Kanobthammakul, S., Nakaguchi, Y., Singkhamanans, K., Yadrak, P., and Sukhumungoon, P. (2014). Virulence characteristics and antimicrobial susceptibility of uropathogens from patients on Phuket Island, Thailand. *Southeast Asian journal of tropical medicine and public health* 45(5)**,** 1090-1098.

Themphachanal, M., Kongpheng, S., Rattanachuay, P., Khianngam, S., Singkhamanan, K., and Sukhumungoon, P. (2015). MOLECULAR CHARACTERIZATION OF VIRULENCE AND ANTIMICROBIAL SUSCEPTIBILITY PROFILES OF UROPATHOGENIC ESCHERICHIA COLI FROM PATIENTS IN A TERTIARY HOSPITAL, SOUTHERN THAILAND. *Southeast Asian journal of tropical medicine and public health* 46(6)**,** 1021-1030.

Thi Quynh Nhi, L., Thanh Tuyen, H., Duc Trung, P., Do Hoang Nhu, T., Duy, P.T., Hao, C.T., et al. (2018). Excess body weight and age associated with the carriage of fluoroquinolone and third-generation cephalosporin resistance genes in commensal Escherichia coli from a cohort of urban Vietnamese children. *Journal of medical microbiology* 67(10)**,** 1457-1466. doi: 10.1099/jmm.0.000820.

Tran, H.H., Ehsani, S., Shibayama, K., Matsui, M., Suzuki, S., Nguyen, M.B., et al. (2015). Common isolation of New Delhi metallo-beta-lactamase 1-producing Enterobacteriaceae in a large surgical hospital in Vietnam. *European journal of clinical microbiology & infectious diseases* 34(6)**,** 1247-1254. doi: 10.1007/s10096-015-2345-6.

Trang, N.H.T., Nga, T.V.T., Campbell, J.I., Hiep, N.T., Farrar, J., Baker, S., et al. (2013). The characterization of ESBL genes in Escherichia coli and Klebsiella pneumoniae causing nosocomial infections in Vietnam. *Journal of infection in developing countries* 7(12)**,** 922-928. doi: 10.3855/jidc.2938.

Trung, N.V., Jamrozy, D., Matamoros, S.b., Carrique-Mas, J.J., Mai, H.H., Hieu, T.Q., et al. (2019). Limited contribution of non-intensive chicken farming to ESBL-producing Escherichia coli colonization in humans in Vietnam: An epidemiological and genomic analysis. *Journal of antimicrobial chemotherapy* 74(3)**,** 561-570. doi: 10.1093/jac/dky506.

Udomsantisuk, N., Nunthapisud, P., Tirawatanapong, T., and Dansuputra, M. (2011). Molecular characterization of extended spectrum beta-lactamase among clinical isolates Escherichia coli and Klebsiella pneumoniae. *Journal of the Medical Association of Thailand= Chotmaihet thangphaet* 94(12)**,** 1504-1512.

van Aartsen, J.J., Moore, C.E., Parry, C.M., Turner, P., Phot, N., Mao, S., et al. (2019). Epidemiology of paediatric gastrointestinal colonisation by extended spectrum cephalosporin-resistant Escherichia coli and Klebsiella pneumoniae isolates in north-west Cambodia. *BMC microbiology* 19(1)**,** 59-59. doi: 10.1186/s12866-019-1431-9.

Vlieghe, E.R., Huang, T.D., Phe, T., Bogaerts, P., Berhin, C., De Smet, B., et al. (2015). Prevalence and distribution of beta-lactamase coding genes in third-generation cephalosporin-resistant Enterobacteriaceae from bloodstream infections in Cambodia. *European journal of clinical microbiology & infectious diseases* 34(6)**,** 1223-1229. doi: 10.1007/s10096-015-2350-9.

Vlieghe, E.R., Phe, T., De Smet, B., Veng, H.C., Kham, C., Lim, K., et al. (2013). Bloodstream infection among adults in Phnom Penh, Cambodia: key pathogens and resistance patterns. *PloS one* 8(3)**,** e59775-e59775. doi: 10.1371/journal.pone.0059775.

Wen-Chien, K., and Stone, G.G. (2020). In vitro activity of ceftazidime–avibactam and comparators against Gram-negative bacterial isolates collected in the Asia–Pacific region as part of the INFORM program (2015–2017). *Annals of Clinical Microbiology and Antimicrobials* 19**,** 1.

Werarak, P., Kiratisin, P., and Thamlikitkul, V. (2010). Hospital-acquired pneumonia and ventilator-associated pneumonia in adults at Siriraj Hospital: etiology, clinical outcomes, and impact of antimicrobial resistance. *J Med Assoc Thai* 93(Suppl 1)**,** S126-138.

Zhao, W.D., Yan, P., Guan, H.N., and Zhang, Q.Z. (2014). Characterization of CTX‐M‐type extended‐spectrum beta‐lactamase in clinical clones of Escherichia coli in Southwest China. *Journal of basic microbiology* 54(3)**,** 247-252. doi: 10.1002/jobm.201200313.

Zheng, R., Zhang, Q., Guo, Y., Feng, Y., Liu, L., Zhang, A., et al. (2016). Outbreak of plasmid-mediated NDM-1-producing Klebsiella pneumoniae ST105 among neonatal patients in Yunnan, China. *Annals of clinical microbiology and antimicrobials* 15(1)**,** 1-8.
